# Decoupling Intrinsic Molecular Efficacy From Platform Effects: An Interpretable Machine Learning Framework for Unbiased Perovskite Passivator Discovery

**DOI:** 10.1002/advs.75486

**Published:** 2026-04-30

**Authors:** Jing Zhang, Ziyuan Li, Shan Gao, Zhen Zhu, Jing Wang, Xiangmei Duan

**Affiliations:** ^1^ School of Physical Science and Technology Ningbo University Ningbo China

**Keywords:** density functional theory, high‐throughput virtual screening, interpretable machine learning, molecular‐platform decoupling, perovskite interface engineering, rational materials discovery

## Abstract

Rational design of interface passivators for perovskite solar cells is hindered by the entanglement of intrinsic molecular efficacy with extrinsic platform‐dependent performance—a confounding factor that obscures true chemical advances. Here, we present a generalizable, interpretable machine learning framework that decouples these effects via an asymptotic saturation model, enabling unbiased discovery of molecules with genuine intrinsic gains. Trained on a curated dataset of 240 experimental entries, our model identifies hydrogen bond acceptor strength and electrostatic potential difference as key descriptors. Guided by these insights, we screened >121 million PubChem compounds using a hierarchical strategy integrating diversity clustering and uncertainty quantification. Five dual‐functional candidates (e.g., TDZ‐S, TZC‐F) are identified, exhibiting superior predicted efficacy (surpassing experimental benchmarks) and high confidence. First‐principles calculations confirm strong chemisorption (E_ads_<−1.7 eV), net electron donation, and optimized interfacial energetics. Crucially, our closed‐loop “data–interpretation–screening–verification” pipeline establishes a transferable paradigm for rational materials design, extendable to other optoelectronic interfaces beyond perovskites.

## Graphical abstract

This study establishes an interpretable machine learning framework that disentangles the intrinsic molecular efficacy of passivators from experimental platform effects—enabling unbiased, high‐throughput discovery of effective perovskite surface modifiers. By integrating a data‐driven asymptotic saturation model with uncertainty‐quantified virtual screening across 121 million molecules, we identify five dual‐functional candidates with strong predicted performance. This closed‐loop strategy transforms materials design from empirical trial‐and‐error to a rational, mechanism‐guided paradigm, accelerating the discovery of next‐generation perovskite solar cell passivators.

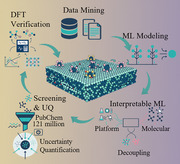

## 1 Introduction

Hybrid perovskites have emerged as the foremost promising materials for next‐generation photovoltaic technologies, propelled by their adjustable bandgaps, cost‐effective fabrication processes, and outstanding optoelectronic characteristics [1, 2, 3, 4]. Recent advancements have pushed the certified efficiencies of single‐junction perovskite solar cells (PSCs) beyond 27% [5], nearing the performance levels of traditional silicon cells. However, the inherent softness of the perovskite lattice leads to the formation of structural defects at both surface and interfaces [6]. These defects typically serve as non‐radiative recombination centers, causing increased voltage losses, reduced device efficiency, and accelerated degradation under operational conditions [7]. Therefore, managing these defects is essential for the commercial realization of PSC technology [8, 9, 10, 11, 12].

Interface passivation has emerged as a pivotal strategy for mitigating these losses, thanks to its localized efficacy and process compatibility [13, 14, 15]. A diverse array of passivating agents, ranging from organic small molecules to polymers, have demonstrated the ability to neutralize deficient Pb^2+^ ions or halide vacancies. This is achieved through Lewis acid‐base interactions, hydrogen bonds, and electrostatic coupling facilitated by functional groups such as ammonium or carboxyl groups [16, 17, 18, 19, 20, 21, 22, 23, 24]. Despite these advancements, the development of efficient passivating agents remains heavily reliant on empirical methods. Yet conducting trial‐and‐error exploration within a vast chemical space is inefficient, and it is difficult to disentangle complex, non‐linear synergies among molecular attributes using traditional single‐variable approaches [25, 26]. Consequently, there is an urgent need for a rational design framework that bridges the gap between chemical structure and device performance. Notably, while single‐functional passivators (e.g., targeting only Lewis acids) have shown promise, they often fall short in addressing the complex, multi‐defect landscape of perovskite surfaces. Developing “dual‐functional” molecules capable of simultaneously neutralizing Lewis acidic and basic defects through synergistic interactions represents a more robust, yet underexplored, avenue for maximizing passivation efficacy [27, 28, 29, 30].

Machine learning (ML) offers a potent tool to tackle these high‐dimensional complexities and now serve as a cutting‐edge instrument for materials discovery, driving a paradigm shift away from traditional trial‐and‐error methods [31, 32, 33, 34]. In the realm of perovskite photovoltaics, ML has been successfully harnessed for a spectrum of applications, including composition optimization, stability forecasting, and absorber layer screening, as well as the recent fine‐screening of small‐molecular passivators utilizing enhanced feature engineering workflows [35, 36, 37, 38, 39, 40, 41, 42]. Nevertheless, a critical gap persists: Many existing ML studies lean heavily on generic molecular fingerprints that lack direct relevance to the specific defect chemistry of perovskites [43, 44, 45, 46]. Moreover, the opaque nature of many models—functioning as “black boxes”—obscures the internal decision logic, thereby limiting their utility for rational, insight‐driven design [47].

Herein, we establish a closed‐loop workflow integrating interpretable ML with theoretical verification to accelerate the discovery of efficient passivators. A feature set tailored for the passivation process was constructed, which was used to train a high‐performance random forest (RF) model (test R^2^ = 0.914). Using SHapley Additive exPlanations (SHAP) analysis [48], we decoded the model to reveal non‐linear, context‐dependent interactions among key molecular features and, crucially, to decouple the intrinsic efficacy of passivators from platform‐dependent baseline effects. Guided by these insights, we screened the PubChem database (>121 million molecules) using a hierarchical strategy incorporating uncertainty quantification to identify five high‐confidence candidates. First‐principles calculations confirmed that these molecules possess strong adsorption, favorable band alignment, and net electron‐donor characteristics. In summary, this work established a complete “data mining—model–interpretation–screening–validation” loop, providing a transferable paradigm for the rational discovery of complex interface materials.

## 2 Results and Discussion

### 2.1 Data‐Driven Framework and Feature Engineering

**FIGURE 1:**
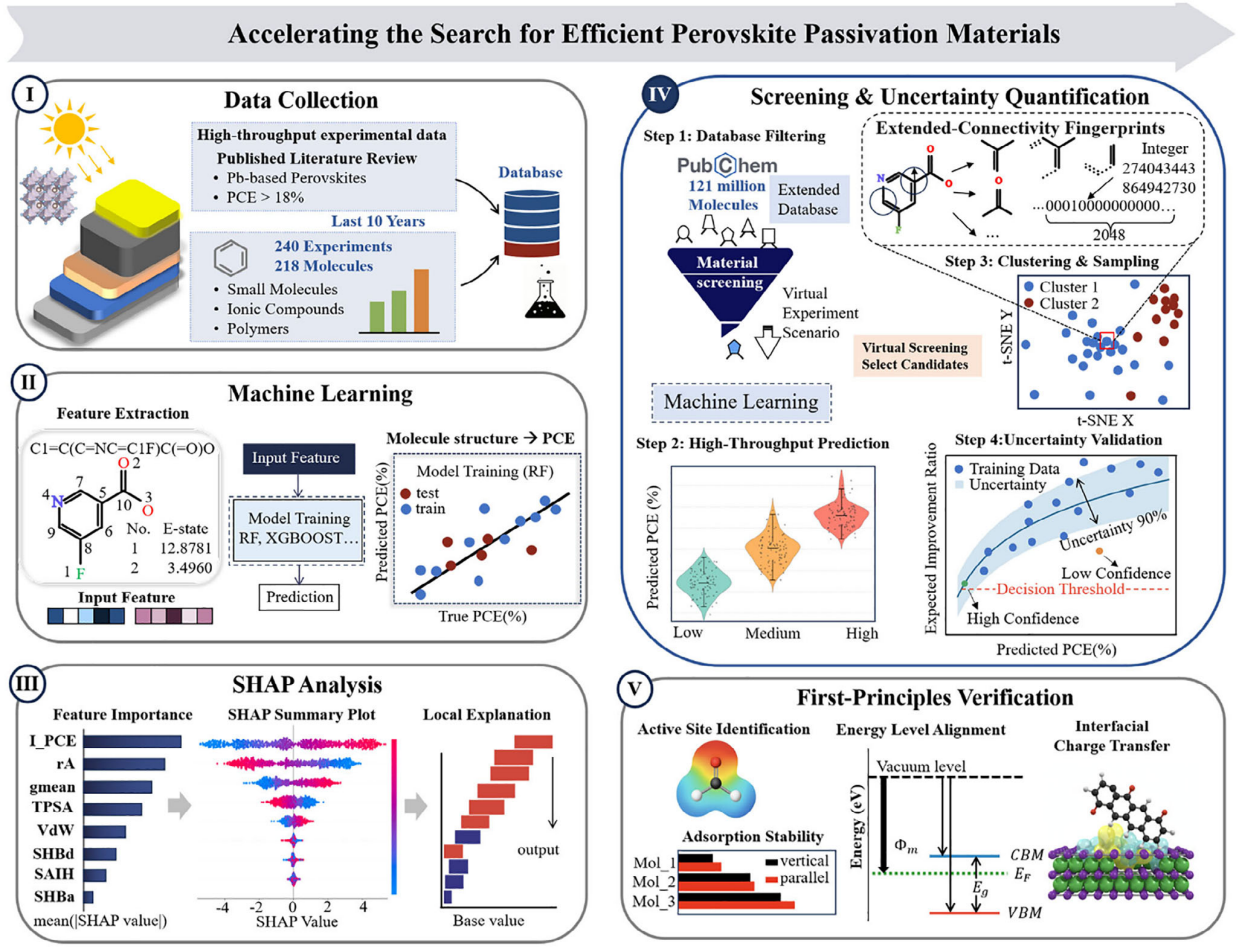
Schematic illustration of the closed‐loop data‐driven workflow developed in this study. The workflow comprises five subsequential steps: (I) Data Collection and Dataset Construction; (II) Machine Learning Modeling; (III) SHAP‐based Interpretability Analysis; (IV) Screening and Uncertainty Quantification; and (V) First‐Principles Verification.

We introduce a meticulously curated data‐driven workflow that synergistically integrates data mining, machine learning, and theoretical verification to accelerate the discovery of efficient perovskite interface passivators. As depicted in Figure 1, the workflow comprises five consecutive steps forming a closed loop. (1) Data Collection: A high‐quality dataset was curated from a systematic literature review spanning the past decade. To target high‐efficiency photovoltaics, we compiled 240 entries involving 218 unique organic passivators (Figure S1 and Table S1), strictly filtering for Pb‐based devices with initial efficiencies >18%. (2) Machine Learning Modeling: Custom features were extracted to train regression models (e.g., Random Forest) for robust structure‐property mapping. (3) Interpretability Analysis: The optimal model was dissected using multi‐level SHAP analysis, progressing from global importance to local attribution for mechanistic insight. (4) Virtual Screening: The model was applied to the PubChem database to identify candidates via hierarchical filtering and uncertainty quantification. (5) First‐Principles Verification: Top candidates were validated using Density Functional Theory (DFT) to probe electrostatic and adsorption properties.

Preliminary statistical analysis reveals that both surface and interface passivation ubiquitously improve photovoltaic parameters, with the power conversion efficiency (PCE) displaying the most pronounced sensitivity to structural variations (Figure S2). Consequently, PCE was selected as the prediction target. To map molecular structures to performance, a 21D feature set was engineered to capture the physicochemical essence of passivation (Table 1). First, initial device efficiency (I_PCE) was introduced as a baseline to decouple intrinsic film quality from passivation effects. This was complemented by the effective cation radius (rA) and precursor molar ratios (A:X, B:X), which critically influence crystallization dynamics and defect formation [49]. Second, considering charge interactions, dipole moments (u, uD) and Gasteiger charge descriptors (gmean, qpmax, qnmax) were incorporated to quantify electronic distribution [50, 51].

**TABLE 1:** Description of the 21D feature set utilized in ML modeling.

	Input features	Feature description
(i)	I_PCE, rA, A:X, B:X	Initial PCE of the control device; Effective radius of the A‐site cation; Molar ratios of A‐site cation or B‐site cation to halide ions in the precursor solution.
(ii)	SHBd, SHBa, SamH, SalH, SarH, SHal	Sum of E‐state indices for: hydrogen bond donors, hydrogen bond acceptors, ammonium group hydrogens, aliphatic hydrogens, aromatic hydrogens, and halogens.
(iii)	u, uD, gmean, qpmax, qnmax	Total dipole moment; Dipole moment difference index; Mean square deviation of Gasteiger charges; Maximum positive and negative Gasteiger partial charges.
(iv)	RBR, EPD, LogP, TPSA, vdW, CPLX	Rotatable bond ratio; Maximum electrostatic potential difference; Octanol‐water partition coefficient Topological polar surface area; Van der Waals volume; Molecular complexity index.

In addition, electro‐topological state indices (E‐state) were computed to assess the defect anchoring capabilities, specifically hydrogen bond donor (SHBd) and acceptor (SHBa) properties [52]. These features quantify chemical adsorption potential by aggregating functional group contributions [53, 54, 55]. To further address steric and interface compatibility, the rotatable bond fraction (RBR) was incorporated to reflect molecular flexibility, while the topological polar surface area (TPSA) and partition coefficient (LogP) were employed to capture polarity and hydrophobicity characteristics [56, 57, 58]. Moreover, the van der Waals volume (vdW) and complexity index (CPLX) were introduced to comprehensively represent steric hindrance and structural intricacy [59]. These customized multidimensional features translate chemical structures of passivators into ML‐readable data, laying the foundation for model training.

### 2.2 Feature Validation and Machine Learning Modeling

To assess the efficacy of the proposed 21D feature set, we employed a Random Forest regression framework as a benchmark, and compared its performance against both a reduced 15‐feature subset and the descriptor ensembles reported in two representative prior studies (see Figure S3 and Table S3). Our full feature set demonstrated superior generalization capability, yielding the highest coefficient of determination on the test set (R^2^ = 0.826 ± 0.030) compared to 0.804 ± 0.042 and 0.797 ± 0.044 for Liu et al.'s and Zhi et al.'s feature sets. Although the simplified 15‐feature subset achieved nearly identical predictive accuracy (R^2^ = 0.826 ± 0.031), the complete 21‐feature representation was retained to preserve essential physicochemical information required for subsequent interpretability analyses [60].

**FIGURE 2:**
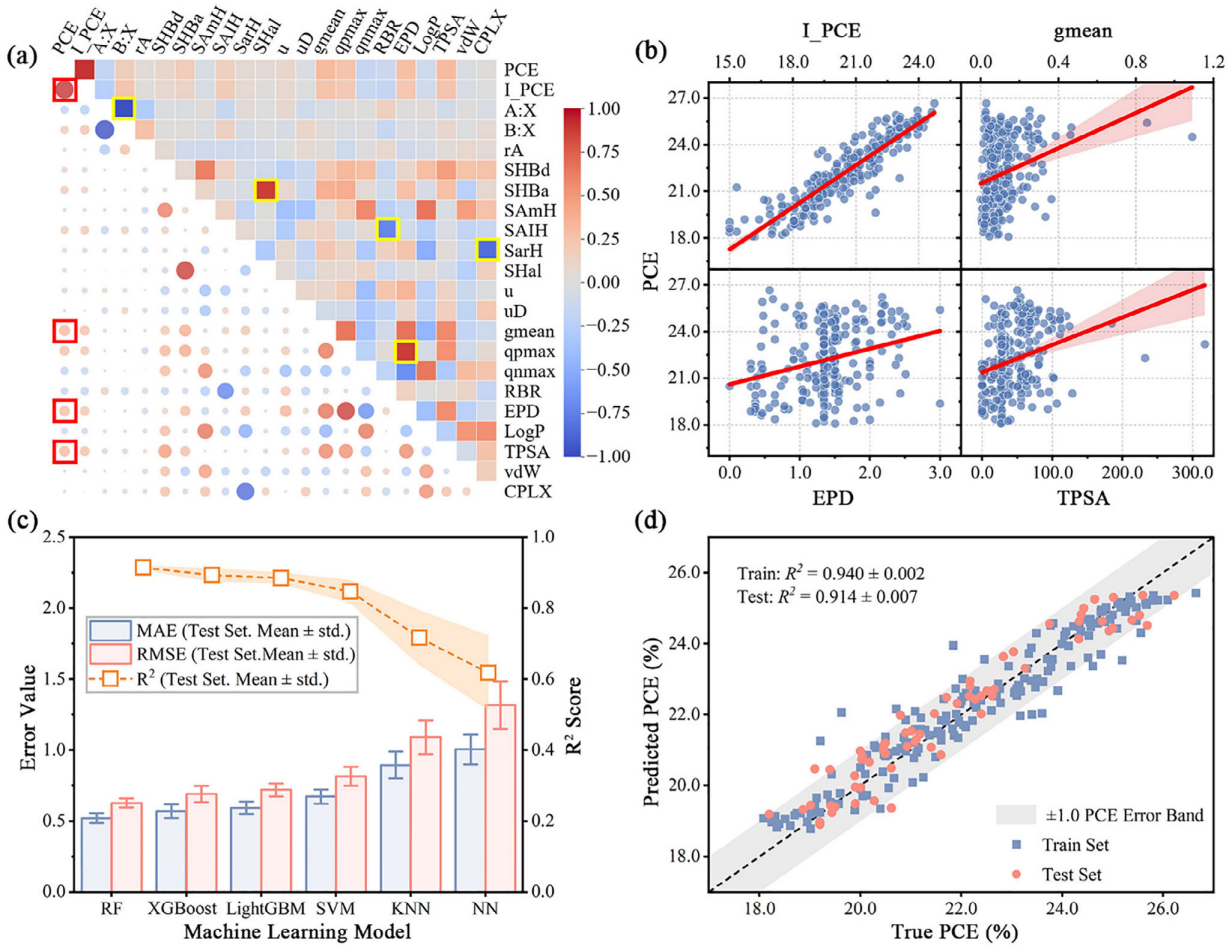
Feature correlation analysis and performance evaluation of machine learning models. (a) Pearson correlation matrix of the 21 input features. Red boxes highlight descriptors that exhibit strong linear correlation with PCE, while yellow boxes indicate high collinearity (∣r∣> 0.7) among features. (b) Linear regression plots showing the relationship between PCE and four key features: I_PCE, gmean, EPD, and TPSA. Shared areas represent 95% confidence intervals. (c) Performance comparison of six regression algorithms on the test set. Error bars denote standard deviations from 10 independent runs. (d) Parity plot comparing predicted vs. experimental PCE values for the optimized RF model. The gray band represents a ±1.0% prediction error band.

Pearson correlation analysis was conducted to evaluate feature redundancy and physical plausibility before ML training (Figure 2a). The majority of descriptor pairs exhibited weak correlations (|r|< 0.7), indicating minimal information overlap. The few instances of strong correlations align well with established physical principles: For instance, the high correlation between the qpmax and EPD (r = 0.87) reflects the intrinsic coupling between localized charge accumulation and the electrostatic potential distribution, while the moderate correlation between SAmH and SAlH (r = 0.66) arises from the shared structural motifs among high‐performing passivators. Notably, precursor stoichiometric ratios (A:X and B:X) displayed stronger linear associations with I‐PCE (|r|≈0.24) than with the final PCE (|r|≈0.17), suggesting that effective passivation reduces device performance sensitivity to processing fluctuations. Furthermore, linear correlation analysis underscores the limited predictive power of individual descriptors. While I‐PCE and final PCE are strongly positive correlated (r = 0.90), other molecular features—such as gmean, TPSA, and EPD—exhibit only modest linear trends (Figure 2b). Intriguingly, the molecular dipole moment (u), despite its well‐documented role in band alignment in experimental studies, shows negligible linear correlation with device performance (Figure 2a) [61]. This apparent inconsistency highlights that passivator efficacy is governed by intricate, nonlinear interactions among multiple physicochemical factors—thereby necessitating ML approaches capable of capturing such higher‐order dependencies [62].

To establish a reliable mapping from molecular descriptors to device performance, six ML models, including RF, XGBoost, LightGBM,s SVM, KNN, and NN, were systematically evaluated. To improve model robustness against outliers, a hard sample mining strategy was implemented, iteratively reweighting data points with large prediction errors during training (Section S2) [63]. As shown in Figure 2c, the RF model consistently outperformed all alternatives, achieving the highest coefficient of determination on the test set (R^2^ = 0.914 ± 0.007) alongside the lowest mean absolute error (MAE) and root‐mean‐square error (RMSE). This represents an ∼12.5% improvement in predictive accuracy compared to the standard training protocol described earlier, corresponding to an absolute R^2^ gain of 0.1—demonstrating RF's superior capacity to model non‐ideal or challenging samples. In contrast, the remaining models exhibited compromised generalization, primarily due to overfitting (e.g., NN, SVM) or underfitting (e.g., KNN), with comprehensive performance metrics provided in the Supporting Information (Figure S4 and Table S4). Consequently, RF was selected as the optimal predictive model for subsequent analyses. Figure 2d further validates the model's fidelity: Predicted PCE values align closely with experimental measurements, with the majority of deviations confined within ±1.0% (shaded region). Residual analysis confirms that the prediction errors are randomly distributed without evident heteroscedasticity (Figure S4f), satisfying key assumptions of regression reliability. A minor systematic overestimation is observed, likely attributable to subtle discrepancies between idealized computational inputs and real‐world experimental conditions; however, this bias remains negligible for high‐throughput candidate screening purposes [64].

### 2.3 Interpretability Analysis to Clarify Molecule‐Platform Coupling

The built‐in importance metric for Random Forest shows the feature ranking, three at the top are I_PCE, rA, and gmean (Figure S5). To uncover the underlying decision logic of the RF model, we performed SHAP analysis [48]. Global SHAP evaluation confirmed that experimental baseline variables—such as I_PCE and rA—exerted dominant influence on model predictions (Figure S6). However, to enable rational molecular design, it is essential to isolate the intrinsic contribution of passivators chemistry from these extrinsic experimental factors. Accordingly, we constructed a dedicated SHAP summary plot focusing exclusively on the 17 molecular features, thereby effectively decoupling passivator‐specific efficacy from platform‐dependent variables—a crucial step for identifying robust chemical drivers independent of fabrication baselines, which was quantitatively validated by a supplementary multi‐stage ablation study (Table S5).

**FIGURE 3:**
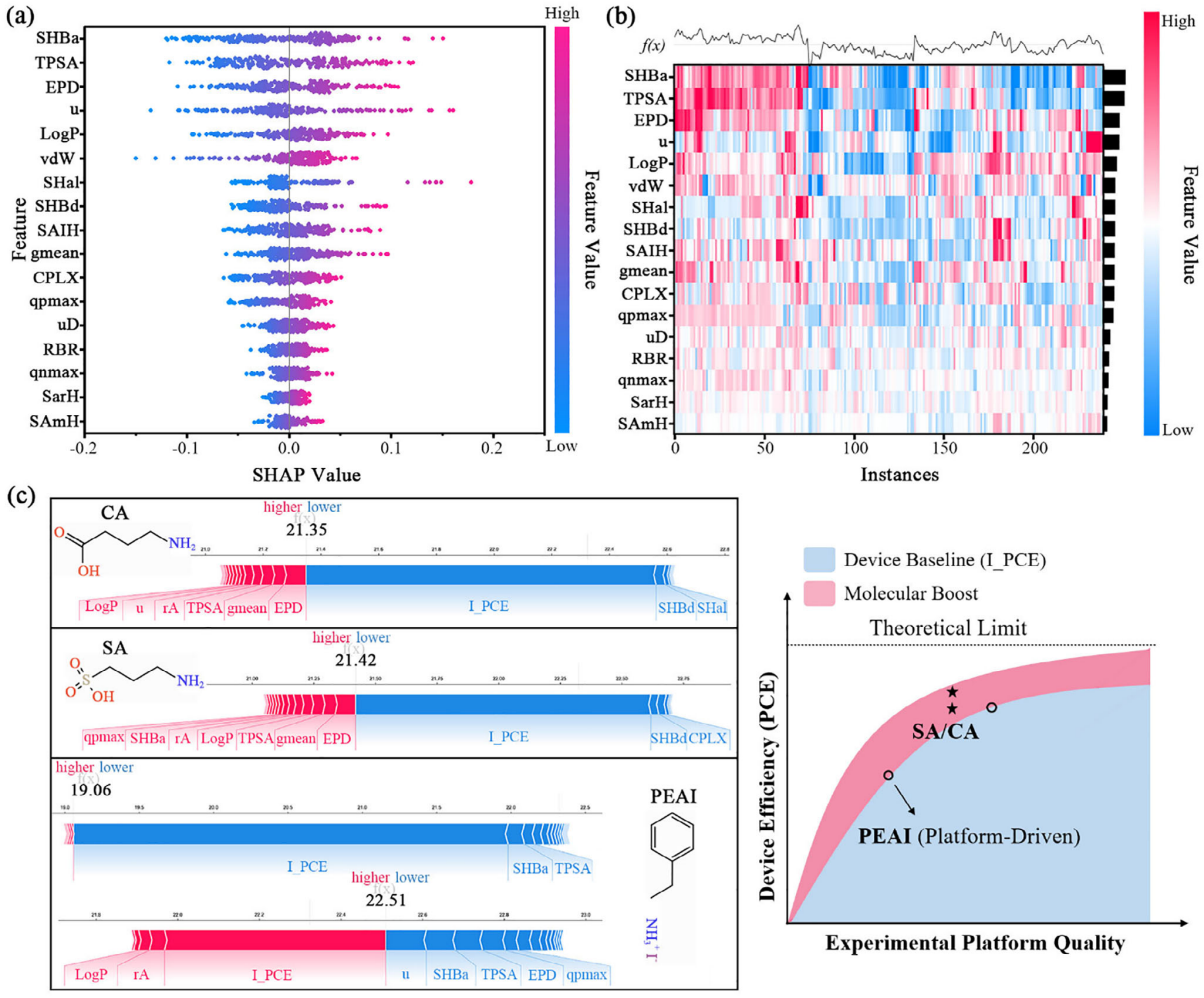
Multi‐level interpretability analysis using SHAP. (a) Summary plot illustrating the global importance and directional impact of the 17 molecular features. (b) Heatmap of feature contributions across all 240 samples, sorted by model output f(x). (c) Local attribution and mechanistic decoupling. Left: SHAP force plots comparing CA vs. SA to elucidate the molecular effect, and PEAI under different baselines conditions to illustrate the platform effect. Right: Schematic representation of an asymptotic saturation model distinguishing the “Intrinsic Molecular Boost” (pink zone, stars) from “Platform‐Driven” efficacy (blue baseline region, circles) relative to the theoretical limit.

As shown in Figure 3a, SHBa emerged as the most impactful relationship: Enhanced hydrogen bond accepting capacity consistently correlates with improved PCE. Similarly, polarity‐associated features (TPSA, EPD, and u) ranked prominently and exhibited uniformly positive SHAP contributions. Conversely, high LogP values were associated with negative SHAP scores, suggesting that excessive hydrophobicity adversely affects device performance, likely due to limited dispersion in polar precursor solvents [65]. Notably, steric properties also played a non‐negligible role: vdW ranked above SHBd in importance and showed a preference for larger molecular volumes. Although SHBd occupies a lower position in the hierarchy, it still displayed a discernible positive trend, suggesting a modest but favorable role in passivation efficacy.

To further investigate how molecular attributes jointly govern performance, we examined non‐linear feature interactions using SHAP dependence plots (Figure S7). Four representative pairs were selected to probe cross‐domain synergies—gmean vs. vdW, SHBa vs. LogP, RBR vs. u, and uD vs. EPD—charge distribution vs. steric bulk, hydrogen bonding vs. hydrophobicity, conformational flexibility vs. polarity, and electrostatic symmetry vs. strength.

The SHAP‐based interaction analysis unveils several actionable molecular design principles. For instance, the SHBa‐LogP pair exhibits a “switch‐like” dependency: The beneficial effect of strong hydrogen bond acceptor capability is contingent upon a moderately hydrophobic environment—highlighting the context‐dependent nature of noncovalent interactions in passivation [66]. Similarly, the interplay between gmean and vdW interaction suggests that optimal performance arises from a delicate balance between electronic heterogeneity and moderate steric bulk, avoiding extremes that could disrupt film morphology or charge transport [67, 68]. Additionally, molecular flexibility (RBR) was found to attenuate the typically detrimental impact of large dipole moments (u), likely by facilitating conformational adaptation at interfaces to minimize energetic penalties [69]. A comprehensive discussion of these cooperative mechanisms is provided in Note S5 and visualized in Figure S7.

Beyond intrinsic molecular features, the model also uncovered delicate dependencies between passivation efficacy and key experimental variables (Figure S8). The influence of parameters such as rA, A:X, and B:X on final PCE is distinctly non‐monotonic and strongly modulated by initial device quality (I_PCE). Specifically, the positive contribution of rA is observed only within a narrow window (2.4–2.6 Å), aligning closely with the ideal range predicted by the Goldschmidt tolerance factor for a stable perovskite lattice [70]. Furthermore, deviations from nominal stoichiometric—particularly Pb‐rich conditions—trend to enhance performance in initially high‐quality films but degrade those with poor baseline characteristics. This dichotomy provides computational support for targeted “Pb‐rich” processing strategies, which appear most effective when applied to already optimized device architectures [71, 72, 73].

To bridge global feature‐level insights with individual predictions, we generated a SHAP heatmap (Figure 3b) that visualizes the contribution of each descriptor across all 240 samples, ordered by predicted PCE. The heatmap demonstrates that high‐performance predictions arise from the concerted positive contributions of multiple features (evident as deep red blocks), reinforcing the notion that effective passivation is governed by synergistic, multivariate effects rather than isolated descriptors.

To further dissect these contributions at the molecular level, SHAP force plots were employed for analyzing representative passivators (Figure 3c, left). A direct comparison between CA and SA reveals that the sulfonyl group (─SO_2_─) in SA substantially enhances the SHAP values of SHBa and qpmax relative to the carboxyl group in CA—thereby accounting for its higher predicted efficiency [74, 75, 76]. In contrast, PEAI exhibits consistently negative or negligible contributions from its molecular features (e.g., u, SHBa), irrespective of device quality. This indicates that its observed PCE improvement stems predominantly from a favorable experimental baseline (I_PCE), underscoring the strong interdependence between passivator chemistry and the underlying device platform [77, 78, 79]. Collectively, these cases studies confirm that model predictions are chemically interpretable and aligned with established structural‐function relationships.

To quantitatively disentangle the roles of molecular design and experimental context, we introduced an asymptotic saturation model (Figure 3c, right). In this framework, the total PCE enhancement follows a non‐linear saturation curve approaching a theoretical upper limit. The blue region represents the performance floor established by the experimental platform (I_PCE), while the superimposed pink zone—termed “Molecular Boost”—captures the additional gain attributable to intrinsic molecular properties. Notably, SA and CA (marked by stars) lie well within the pink zone, confirming that their functional groups (e.g., ‐SO_2‐_) confer genuine chemical advantages beyond platform effects. Conversely, PEAI (circles) closely adheres to the baseline boundary, consistent with its weak SHAP feature scores and implying that its efficacy is largely platform‐dependent. High final PCE derives primarily from the high‐quality platform rather than intrinsic chemical. This decomposition highlights a critical design principle: Future high‐impact passivators must be engineered to actively penetrate the “Molecular Boost” regime, rather than relying on fortuitous compatibility with high‐performing platforms. By explicitly separating these contributions, our framework ensures the unbiased discovery of molecules with true intrinsic gains, overcoming the confounding factors that often obscure chemical advances in literature.

### 2.4 Large‐Scale Virtual Screening and Candidate Discovery

**FIGURE 4:**
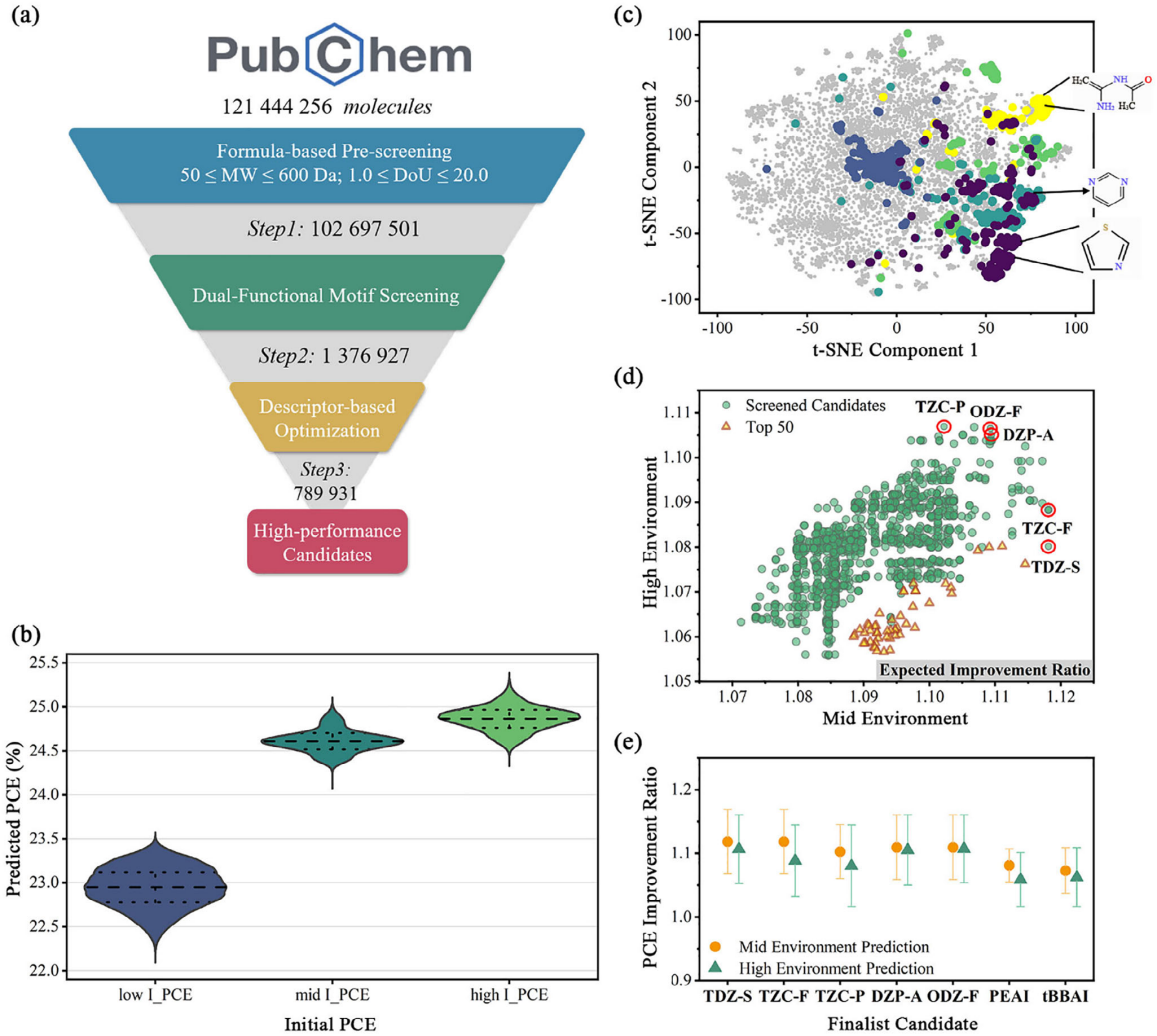
High‐throughput virtual screening and discovery of candidate passivators. (a) Schematic of the hierarchical screening workflow. The pipeline reduces the chemical space from over 121 million PubChem entries to a refined library of high‐potential candidates through a series of filters. (b) Violin plots of predicted PCE distributions. These plots show the predicted PCE distributions for the refined library across three virtual scenarios. (c) t‐SNE visualization of the chemical space. The plot visualizes the chemical diversity of elite candidates based on ECFP fingerprints. (d) Expected PCE improvement ratios. The plot compares the expected improvement ratios for screened candidates (green dots) vs. training set molecules (orange crosses), with the top five finalists highlighted in red circles. (e) Performance comparison and uncertainty quantification. This panel evaluates the performance and uncertainty of the five finalists against experimental benchmarks (PEAI, tBBAI) under medium and high‐performance conditions.

Leveraging the validated predictive model and the mechanistic insights derived from SHAP analysis, we implemented a scalable framework for high‐throughput molecular discovery. A hierarchical virtual screening pipeline was developed to interrogate the PubChem database—encompassing over 121 million compounds—for promising passivator candidates (Figure 4a). The workflow comprised three sequential filtering steps. Step 1 employed computationally lightweight, formula‐based criteria—including MW (molecular weight), DoU (degree of unsaturation), elemental ratios—to exclude thermally unstable, synthetically intractable, or overly complex structures. This initial curation reduced the candidate pool to approximately 102 million molecules. In Step 2, we introduced a chemically informed “dual‐functional” motif filter based on SMILES pattern matching. Candidates were required to simultaneously harbor both electron‐donating groups (e.g., H‐bond donor) and electron‐accepting moieties (e.g., Lewis base). This design principle targets the coexistence of Lewis acidic sites (e.g., undercoordinated Pb^2+^) and Lewis basic defects (e.g., halide vacancies) commonly found on perovskite surfaces, thereby enabling multidentate, synergistic passivation through cooperative binding [80, 81, 82]. Application of this constraint narrowed the library to 1.38 million structurally plausible candidates. Step 3 incorporated feature‐based physicochemical optimization. Key 2D molecular features—related to polarity (TPSA), LogP, steric size, conformational flexibility, and topological complexity—were computed and subjected to empirically grounded bounds. These constraints, while avoiding the computational overhead of the full 21D feature set, were directly informed by trends identified in our SHAP interpretability analysis. Analogous to “drug‐likeness” rules in medicinal chemistry, these filters ensure favorable solution processability, interfacial compatibility, and film‐forming behavior [83]. This three‐tiered filtration strategy ultimately yielded a refined library of 789,931 high‐potential passivators suitable for downstream predictive ranking. Full screening criteria and parameter thresholds are detailed in Note S6.

Using the trained RF model, we evaluated the performance of the curated library under three virtual experimental scenarios. All scenarios have identical system‐level parameters—namely rA = 2.48, A:X = 0.34, B:X = 0.33—extracted from high‐performance samples in our dataset, while varying the initial baseline quality (I_PCE) to reflect the 60th (21.09%), 80th (22.60%), and 95th (23.51%) percentiles of the baseline distribution. As shown in Figure 4b, the predicted PCE distribution exhibited clear sensitivity to I‐PCE: Higher‐quality starting platforms not only shifted the entire distribution upward but also reduced its variance, indicating more consistent and reliable passivation outcomes on optimized substrates.

To prioritize candidates for downstream validation, we devised a selection strategy that jointly optimizes predictive performance and structural diversity. From each scenario, the top 5000 high‐scoring molecules were extracted. Molecules appearing in all three scenario‐specific top lists were designated as core candidates, reflecting robustness across varying baseline conditions. The remaining 12 888 unique high‐performers—present in one or two scenarios—were subjected to diversity‐aware filtering to avoid chemical redundancy. Structurally similarity was quantified using Extended Connectivity Fingerprints (ECFPs), and the Butina clustering algorithm was applied to partition the set into structurally coherent groups [84, 85, 86]. t‐SNE embedding of the fingerprint space (Figure 4c) reveals that high‐performing candidates populate multiple well‐separated clusters, underscoring significant scaffolds diversity. The five largest clusters were identified, and their representative molecular scaffolds are highlighted for clarity [87, 88]. From each cluster, the top 5 molecules were selected based on a weighted ranking score that integrates predicted PCE, SHAP‐based feature favorability, and synthetic accessibility (see Note S8). This approach ensures broad coverage of chemical space while mitigating overrepresentation of any single structural motif.

The 1201 structurally diverse representative molecules were prioritized using the “Expected Improvement Ratio” (EIR), defined as the predicted PCE divided by I_PCE. As shown in Figure 4d, their distribution across medium‐ and high‐performance scenarios reveals that the newly screened candidates (green dots) consistently occupy regions of higher potential compared to re‐evaluated known passivators (orange crosses), thereby validating the efficacy of the discovery workflow. Nevertheless, reliance on point predictions alone entails inherent risk due to model uncertainty [89, 90]. Without uncertainty filtering, ∼40% of the top‐100 candidates exhibit wide prediction intervals (> ±0.05 EIR width), indicating a high risk of overestimation and potential false positives. To address this, a Random Forest Quantile Regression model was trained to estimate the 90% prediction interval for the EIR (see Note S7). Candidates were then filtered using a dual criterion: High expected improvement coupled with low predictive uncertainty (i.e., narrow confidence intervals). This strategy yielded five top‐performing molecules, highlighting as red circles in Figure 4d.

Figure 4e presents a performance comparison between the five‐top ranked candidates and established experimental benchmarks—PEAI and tBBAI. Across all virtual scenarios, the selected candidates consistently deliver an improvement ratio exceeding 1.08, surpassing both reference passivators in predicted efficacy. For clarity and conciseness, each candidate is labeled according to its core heterocyclic scaffold: TDZ‐S (thiadiazolidine dioxide), TZC‐F and TZC‐P (thiazolidine derivatives functionalized with difluoro and pivaloyl groups, respectively), DZP‐A (diazepane‐based architecture), and ODZ‐F (fluorinated oxadiazole). Complete IUPAC names, chemical structures, and associated descriptors are provided in Table S6. Importantly, automated retrosynthesis analysis via ChemAIRS confirms that all five finalists are either from commercially available precursors or accessible in 2–4 synthetic steps (see Note S8 and Figure S10), underscoring their experimental feasibility. As the principal output of our data‐driven discovery pipeline, these molecules represent high‐value, chemical diverse leads for synthesis and device‐level validation.

### 2.5 First‐Principles Verification of Atomistic Mechanisms

**FIGURE 5:**
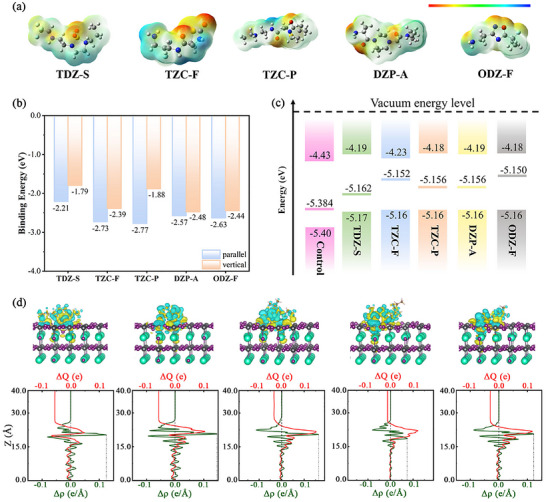
First‐principles validation of the finalist candidates. (a) Electrostatic potential (ESP) maps are mapped onto the electron density isosurface (0.002 a.u.). (b) Calculated E_ads_ for the five candidates on the β‐CsPbI_3_(001) surface, comparing parallel and vertical configurations. (c) Energy level alignment diagram showing the shift in Fermi level (E_F_) and work function (Φ) upon molecule adsorption relative to the pristine control. (d) Differential charge density analysis for the most stable adsorption configurations. Upper panels: 3D isosurfaces (yellow: accumulation; cyan: depletion). Lower panels: Planar‐averaged charge density difference (Δρ, green curves) and integrated charge displacement (ΔQ, red curves) along the *z*‐axis.

To uncover the atomistic and electronic underpinnings of the predicted performance, we carried out first‐principles calculations (see Note S9). We began by analyzing the electrostatic potential (ESP) mapped onto the electron density isosurface, as shown in Figure 5a. A consistent electronic motif emerged across all five top candidates: Pronounced charge inhomogeneity featuring spatially separated electron‐rich (red) and electron‐deficient (blue) regions. This intrinsic polarization aligns directly with SHAP‐based insights that identified high gmean and strong EPD as critical performance drivers.

Notably, strong negative ESP minima were localized on electronegative functional groups—such as sulfonyl (─SO_2_─) and carbonyl (─C═O) oxygen atoms in TDZ‐S and TZC‐F. These sites serve as potent Lewis bases and hydrogen bond acceptors, corroborating the high SHAP importance assigned to SHBa. Analogous electron‐rich centers were observed on amide oxygens and heterocyclic atoms in DZP‐A (a piperazine derivative) and ODZ‐F (an oxadiazole derivative), reinforcing their capacity for anion coordination. Conversely, pronounced positive ESP maxima were found on amine or amide protons (─NH), particularly in TDZ‐S and DZP‐A. These electropositive sites function as hydrogen bond donors, enabling favorable interactions with surface halide vacancies—a role consistent with the positive (though secondary) contribution of SHBd in our interpretability analysis. These electronic features establish a “dual‐functional” molecule architecture: Each candidate simultaneously presents complementary Lewis acidic and basic sites, enabling cooperative, multi‐point passivation of coexisting Pb^2+^ halide defects. This atomistic picture provides a mechanistic foundation for the superior performance predicted by our ML model, confirming that the high predicted efficacy stems from intrinsic molecular capabilities rather than platform dependencies.

The binding affinity of the top candidates to the β‐CsPbI_3_(001) surface—terminated with PbI_2_ layer—was quantified via adsorption energies (E_ads_). Multiple adsorption configurations were evaluated, with particular focus on energetically favorable “parallel” and “vertical” orientations (Figure 5b). All five molecules exhibited strong chemisorption, with E_ads_ values below −1.7 eV—significantly more exothermic than typical physisorption thresholds (< 0.5 eV). Notably, TZC‐F and DZP‐A achieved exceptionally stable binding, with E_ads_ being −2.73 and −2.63 eV, respectively, underscoring their robust interfacial anchoring capability.

This chemisorption profoundly modulates the surface electronic structure. As shown in the energy level alignment diagram (Figure 5c), molecular adsorption induces a pronounced shift in the Fermi level (E_F_) and a marked reduction in the work function (Φ). For instance, TZC‐F lowers Φ from 5.38 eV (pristine surface) to 5.15 eV. Such a reduction improves band alignment with a common electron transport layer, thereby diminishing the interfacial barrier and promoting efficient electron extraction [50]. Concurrently, the positions of the valence band maximum (VBM) and conduction band minimum (CBM) are subtly shifted upon adsorption, potentially enhancing interfacial carrier dynamics and reducing recombination losses [91, 92].

To elucidate the bonding mechanism, we performed differential charge density analysis (Figure 5d). The 3D isosurfaces (upper panels) visualize interfacial charge redistribution: Yellow regions denote electron accumulation, while cyan regions indicate depletion. Clear charge transfer channels emerge across all systems. Taking TZC‐F as an example, significant electron accumulation occurs between the sulfonyl oxygen and undercoordinated surface Pb atoms, accompanied by electron depletion around Pb sites—characteristic of coordinative bonding. This confirms that the ESP‐identified electron‐rich centers serve as active anchoring sites for Lewis acidic Pb^2+^. Analogous charge transfer patterns are consistently observed for the sulfonyl group in TDZ‐S, the carbonyl (─C═O) moiety in TZC‐P, and the amide oxygen in DZP‐A, collectively validating the dual‐functional design principle at the atomic scale.

Quantitative analysis of the planar‐averaged differential charge density (Δρ, green curves) revealed pronounced interfacial oscillations (0.1−0.2 e/Å^3^), indicative of strong chemical bonding at the molecule‐perovskite interface. Complementarily, integrated charge displacement profiles (ΔQ, red curves) reveal that all five candidates exhibit negative plateau values in ΔQ (−0.05 to −0.15 *e*), conforming their role as net electron donors to the perovskite lattice. This unidirectional charge transfer directly passivates electron‐deficient surface defects—particularly undercoordinated Pb^2+^ ions—thereby neutralizing deep‐level trap states that drive non‐radiative recombination centers [14, 93]. Critically, this consistent electron‐donating behavior emerged organically from our data‐driven discovery pipeline: The machine learning framework converged on molecules with this specific electronic character without explicit quantum‐chemical constraints, demonstrating its capacity to uncover latent structure‐function relationships and identify intrinsic passivation mechanisms. The resulting Lewis acid–base interaction—where electron‐rich functional groups on the passivators donate charge to Lewis acidic Pb^2+^ sites—forms robust coordinative bonds that account for both the high adsorption energies and the favorable interfacial energy alignment. These atomistic insights provide a mechanism foundation for the experimental prioritization of these candidates for synthesis and device integration.

## 3 Conclusions

In conclusion, we establish a generalizable, interpretable machine learning framework that decouple intrinsic molecular efficacy from platform‐dependent performance—a long standard challenge in rational passivator design for perovskite interface. Built on 240 experimentally validated entries and a physically informed 21D feature set, our hard sample mining strategy Random Forest model achieves high predictive accuracy (R^2^ = 0.914). SHAP‐based interpretability analysis reveals hydrogen bond acceptor strength (SHBa and electrostatic potential difference (EPD) as key molecular determinants for intrinsic passivation efficacy. Crucially, we introduced an asymptotic saturation model that quantitatively separates “Platform‐Driven” baseline performance from the “Intrinsic Molecular Boost.” This analysis demonstrates that conventional agents like PEAI largely mirror substrate quality, whereas sulfonic (SA) and cinnamic acid (CA) deliver genuine chemical enhancements that push device efficiency toward its theoretical limit—the “pink zone.” Together with a newly identified “hydrophobicity‐gated” interaction mechanism, these insights yield actionable, substrate‐independent design rules.

Guided by these principles, we perform hierarchical virtual screening across >121 million molecules in PubChem, integrating dual‐functional motif constraints, SHAP‐informed filters, diversity clustering, and uncertainty‐aware ranking. This yields five high‐priority candidates—TDZ‐S, TZC‐F, TZC‐P, DZP‐A, and ODZ‐F—All predicted to enhance the device performance ratio greater than 1.08 relative to state‐of‐art benchmarks. First‐principles calculations validate their “dual‐functional” architectures: Spatially separated Lewis basic/acidic sites enable strong chemisorption (E_ads_ < −1.7 eV), net electron donation (–0.05 to –0.15 *e*), and effective passivation of undercoordinated Pb^2+^ defects, while concurrently optimizing interfacial energy alignment for efficient charge extraction.

Beyond delivering synthetically accessible leads, this work establishes a close‐loop “data–interpretation–screening–verification” paradigm for materials discovery. It demonstrates how physically grounded, interpretable ML can unravel complex structure‐property relationships and accelerate rational design. By reparameterizing descriptors and adapting datasets to specific interfacial mechanisms without substantial redesign of the core ML pipeline, this framework could be extended to diverse engineering challenges, such as buried transport layer modifiers and 2D perovskite modulation, as well as other optoelectronic systems, including OLEDs and quantum dot devices.

## Funding

This work was supported by the National Natural Science Foundation of China (Grant No. 12204256, No. 12374061). The computations were supported by high performance computing center at Ningbo University.

## Conflicts of Interest

The authors declare no conflicts of interest.

## Supporting information




**Supporting File**: advs75486‐sup‐0001‐SuppMat.docx.

## Data Availability

The data that support the findings of this study are available from the corresponding author upon reasonable request.

## References

[1] M. Cai , Y. Wu , H. Chen , X. Yang , Y. Qiang , and L. Han , “Cost‐Performance Analysis of Perovskite Solar Modules,” Advanced Science 4 (2017): 1600269, 10.1002/advs.201600269. 28105403 PMC5238749

[2] M. Li , J. Luo , J. He , et al., “Bandgap Engineering Based on a‐Site Ions Tuning in Tin Halide Perovskite,” Small 21 (2025): 2409546, 10.1002/smll.202409546. 39828638

[3] J. Guo , B. Wang , D. Lu , et al., “Ultralong Carrier Lifetime Exceeding 20 µs in Lead Halide Perovskite Film Enable Efficient Solar Cells,” Advanced Materials 35 (2023): 2212126, 10.1002/adma.202212126. 37163976

[4] W. Chu , W. A. Saidi , J. Zhao , and O. V. Prezhdo , “Soft Lattice and Defect Covalency Rationalize Tolerance of β‐CsPbI_3_ Perovskite Solar Cells to Native Defects,” Angewandte Chemie 132 (2020): 6497–6503, 10.1002/ange.201915702. 31958363

[5] Z. Xiong , Q. Zhang , K. Cai , et al., “Homogenized Chlorine Distribution for >27% Power Conversion Efficiency in Perovskite Solar Cells,” Science 390 (2025): 638–642, 10.1126/science.adw8780. 41196999

[6] Z.‐W. Gao , Y. Wang , and W. C. H. Choy , “Buried Interface Modification in Perovskite Solar Cells: A Materials Perspective,” Advanced Energy Materials 12 (2022): 2104030, 10.1002/aenm.202104030.

[7] G. O. Odunmbaku , S. Chen , B. Guo , et al., “Recombination Pathways in Perovskite Solar Cells,” Advanced Materials Interfaces 9 (2022): 2102137, 10.1002/admi.202102137.

[8] M. I. Saidaminov , J. Kim , A. Jain , et al., “Suppression of Atomic Vacancies via Incorporation of Isovalent Small Ions to Increase the Stability of Halide Perovskite Solar Cells in Ambient Air,” Nature Energy 3 (2018): 648–654, 10.1038/s41560-018-0192-2.

[9] N. Li , S. Tao , Y. Chen , et al., “Cation and Anion Immobilization Through Chemical Bonding Enhancement With Fluorides for Stable Halide Perovskite Solar Cells,” Nature Energy 4 (2019): 408–415, 10.1038/s41560-019-0382-6.

[10] C. Zhang , Y. Wang , X. Lin , et al., “Effects of a Site Doping on the Crystallization of Perovskite Films,” Journal of Materials Chemistry A 9 (2021): 1372–1394, 10.1039/D0TA08656H.

[11] P. Delugas , C. Caddeo , A. Filippetti , and A. Mattoni , “Thermally Activated Point Defect Diffusion in Methylammonium Lead Trihalide: Anisotropic and Ultrahigh Mobility of Iodine,” The Journal of Physical Chemistry Letters 7 (2016): 2356–2361, 10.1021/acs.jpclett.6b00963. 27237630

[12] X. Meng , J. Lin , X. Liu , et al., “Highly Stable and Efficient FASnI_3_‐Based Perovskite Solar Cells by Introducing Hydrogen Bonding,” Advanced Materials 31 (2019): 1903721, 10.1002/adma.201903721. 31495977

[13] G. Yang , Z. Ren , K. Liu , et al., “Stable and Low‐Photovoltage‐Loss Perovskite Solar Cells by Multifunctional Passivation,” Nature Photonics 15 (2021): 681–689, 10.1038/s41566-021-00829-4.

[14] M. Azam , Y. Ma , B. Zhang , et al., “Tailoring Pyridine Bridged Chalcogen‐Concave Molecules for Defects Passivation Enables Efficient and Stable Perovskite Solar Cells,” Nature Communications 16 (2025): 602, 10.1038/s41467-025-55815-z. PMC11724979 39799125

[15] M. Wang , Z. Shi , C. Fei , et al., “Ammonium Cations With High pKa in Perovskite Solar Cells for Improved High‐Temperature Photostability,” Nature Energy 8 (2023): 1229–1239, 10.1038/s41560-023-01362-0.

[16] S. Yang , S. Chen , E. Mosconi , et al., “Stabilizing Halide Perovskite Surfaces for Solar Cell Operation With Wide‐Bandgap Lead Oxysalts,” Science 365 (2019): 473–478, 10.1126/science.aax3294. 31371610

[17] C. Chen , Y. Xu , S. Wu , et al., “CaI_2_: A More Effective Passivator of Perovskite Films Than PbI_2_ for High Efficiency and Long‐Term Stability of Perovskite Solar Cells,” Journal of Materials Chemistry A 6 (2018): 7903–7912, 10.1039/C7TA11280G.

[18] Y. Yang , H. Chen , C. Liu , et al., “Amidination of Ligands for Chemical and Field‐Effect Passivation Stabilizes Perovskite Solar Cells,” Science 386 (2024): 898–902, 10.1126/science.adr2091. 39571031

[19] S. Tan , B. Yu , Y. Cui , et al., “Temperature‐Reliable Low‐Dimensional Perovskites Passivated Black‐Phase CsPbI_3_ Toward Stable and Efficient Photovoltaics,” Angewandte Chemie International Edition 61 (2022): 202201300, 10.1002/anie.202201300. 35243747

[20] J. Wang , H. Ma , A. Wang , et al., “An Ammonium‐Pseudohalide Ion Pair for Synergistic Passivating Surfaces in FAPbI_3_ Perovskite Solar Cells,” Matter 5 (2022): 2209–2224, 10.1016/j.matt.2022.04.006.

[21] Q. Wang , W. Tang , Y. Chen , W. Qiu , Y. Wu , and Q. Peng , “Over 25% Efficiency and Stable Bromine‐Free RbCsFAMA‐Based Quadruple Cation Perovskite Solar Cells Enabled by an Aromatic Zwitterion,” Journal of Materials Chemistry A 11 (2023): 1170–1179, 10.1039/D2TA08878A.

[22] D. Bi , C. Yi , J. Luo , et al., “Polymer‐Templated Nucleation and Crystal Growth of Perovskite Films for Solar Cells With Efficiency Greater Than 21%,” Nature Energy 1 (2016): 16142, 10.1038/nenergy.2016.142.

[23] T.‐H. Han , J.‐W. Lee , C. Choi , et al., “Perovskite‐Polymer Composite Cross‐Linker Approach for Highly‐Stable and Efficient Perovskite Solar Cells,” Nature Communications 10 (2019): 520, 10.1038/s41467-019-08455-z. PMC6355927 30705276

[24] M. Abdi‐Jalebi , Z. Andaji‐Garmaroudi , S. Cacovich , et al., “Maximizing and Stabilizing Luminescence From Halide Perovskites With Potassium Passivation,” Nature 555 (2018): 497–501, 10.1038/nature25989. 29565365

[25] Y. Jiang , L. Ming , Z. Liu , S. Li , L. Chen , and X. Huo , “Synergistic Defect Passivation and Energy‐Level Optimization Toward High‐Efficiency HTL‐Free Carbon‐Based CsPbI_2.75_Br_0.25_ Perovskite Solar Cells,” Chemical Engineering Journal 519 (2025): 165695, 10.1016/j.cej.2025.165695.

[26] K. Rakstys , J. Xia , Y. Zhang , et al., “Steric Hindrance Driven Passivating Cations for Stable Perovskite Solar Cells With an Efficiency Over 24%,” Journal of Materials Chemistry A 12 (2024): 1422–1428, 10.1039/D3TA03423B.

[27] S. He , M. Li , J. Ding , Z. Zhang , and C. Chen , “Multisite Anchoring Strategy of Rationally Designed Molecular Passivator for Achieving Efficient and Stable Perovskite Solar Cells,” Nano Letters 25 (2025): 14195–14203, 10.1021/acs.nanolett.5c03860. 40955914

[28] M. H. Lu , J. K. Ding , Q. X. Ma , et al., “Dual‐Site Passivation by Heterocycle Functionalized Amidinium Cations Toward High‐Performance Inverted Perovskite Solar Cells and Modules,” Energy & Environmental Science 18 (2025): 5973–5984, 10.1039/D5EE00524H.

[29] Y. Wang , Z. Feng , Y. Zhang , et al., “Bifunctional Ligand‐Mediated Dual‐Site Passivation Enables High‐Performance Perovskite Solar Cells With Efficiency Exceeding 26%,” Advanced Functional Materials 36 (2026): 10458, 10.1002/adfm.202510458.

[30] J. Guo , Z. Liu , C. Zhao , et al., “Synergistic Passivation of Defects With a Multifunctional Additive for Perovskite Solar Cells,” Applied Physics Letters 127 (2025): 043902, 10.1063/5.0281855.

[31] Z. Wang , H. Zhang , J. Ren , et al., “Predicting Adsorption Ability of Adsorbents at Arbitrary Sites for Pollutants Using Deep Transfer Learning,” npj Computational Materials 7 (2021): 1–9, 10.1038/s41524-021-00494-9.

[32] Y. Han , I. Ali , Z. Wang , et al., “Machine Learning Accelerates Quantum Mechanics Predictions of Molecular Crystals,” Physics Reports 934 (2021): 1–71, 10.1016/j.physrep.2021.08.002.

[33] Z. Wang , Y. Han , X. Lin , J. Cai , S. Wu , and J. Li , “An Ensemble Learning Platform for the Large‐Scale Exploration of New Double Perovskites,” ACS Applied Materials & Interfaces 14 (2022): 717–725, 10.1021/acsami.1c18477. 34967594

[34] V. de la Asunción‐Nadal , C. I. Sprague , B. Guijarro‐Berdiñas , U. B. Cappel , and A. García‐Fernández , “Machine Learning for Perovskite Solar Cells: A Comprehensive Review on Opportunities and Challenges for Materials Scientists,” EES Solar 1 (2025): 927–957, 10.1039/D5EL00041F.

[35] Y. Jiang , Y. Zheng , J. Li , et al., “Data‐Driven Insights to Tackle Photo‐Induced Phase Segregation for Mixed‐Halide Perovskite Solar Cells,” Chemical Engineering Journal 520 (2025): 165704, 10.1016/j.cej.2025.165704.

[36] X. Cai , Y. Zhang , Z. Shi , et al., “Discovery of Lead‐Free Perovskites for High‐Performance Solar Cells via Machine Learning: Ultrabroadband Absorption, Low Radiative Combination, and Enhanced Thermal Conductivities,” Advanced Science 9 (2022): 2103648, 10.1002/advs.202103648. 34904393 PMC8811845

[37] X. Zhang , B. Ding , Y. Wang , et al., “Machine Learning for Screening Small Molecules as Passivation Materials for Enhanced Perovskite Solar Cells,” Advanced Functional Materials 34 (2024): 2314529, 10.1002/adfm.202314529.

[38] Q. Lou , J. Wang , Z. Nie , et al., “Prediction and Fine Screening of Small Molecular Passivation Materials for High‐Efficiency Perovskite Solar Cells via an Enhanced Machine Learning Workflow,” Advanced Functional Materials 35 (2025): 2511549, 10.1002/adfm.202511549.

[39] Y. Pu , Z. Dai , Y. Zhou , et al., “Data‐Driven Molecular Encoding for Efficient Screening of Organic Additives in Perovskite Solar Cells,” Advanced Functional Materials 36 (2026): 06672, 10.1002/adfm.202506672.

[40] Q. Zhang , H. Wang , Q. Zhao , et al., “Machine‐Learning‐Assisted Design of Buried‐Interface Engineering Materials for High‐Efficiency and Stable Perovskite Solar Cells,” ACS Energy Letters 9 (2024): 5924–5934, 10.1021/acsenergylett.4c02610.

[41] C. Zhi , S. Wang , S. Sun , et al., “Machine‐Learning‐Assisted Screening of Interface Passivation Materials for Perovskite Solar Cells,” ACS Energy Letters 8 (2023): 1424–1433, 10.1021/acsenergylett.2c02818.

[42] Q. Lou , J. Wang , Z. Nie , et al., “Prediction and Fine Screening of Small Molecular Passivation Materials for High‐Efficiency Perovskite Solar Cells via an Enhanced Machine Learning Workflow,” Advanced Functional Materials 35 (2025): 11549, 10.1002/adfm.202511549.

[43] A. Ghosh and S. Ghosh , “Mapping Causal Pathways With Structural Modes Fingerprint for Perovskite Oxides,” Machine Learning: Science and Technology 5 (2024): 045014, 10.1088/2632-2153/ad7d5e.

[44] L. Williams , A. Mukherjee , and K. Rajan , “Deep Learning Based Prediction of Perovskite Lattice Parameters From Hirshfeld Surface Fingerprints,” The Journal of Physical Chemistry Letters 11 (2020): 7462–7468, 10.1021/acs.jpclett.0c02201. 32841568

[45] X. Li and D. Fourches , “SMILES Pair Encoding: A Data‐Driven Substructure Tokenization Algorithm for Deep Learning,” Journal of Chemical Information and Modeling 61 (2021): 1560–1569, 10.1021/acs.jcim.0c01127. 33715361

[46] I. Mendolia , S. Contino , G. De Simone , U. Perricone , and R. Pirrone , “EMBER—Embedding Multiple Molecular Fingerprints for Virtual Screening,” International Journal of Molecular Sciences 23 (2022): 2156, 10.3390/ijms23042156. 35216273 PMC8877815

[47] F. Oviedo , J. L. Ferres , T. Buonassisi , and K. T. Butler , “Interpretable and Explainable Machine Learning for Materials Science and Chemistry,” Accounts of Materials Research 3 (2022): 597–607, 10.1021/accountsmr.1c00244.

[48] E. Štrumbelj and I. Kononenko , “Explaining Prediction Models and Individual Predictions With Feature Contributions,” Knowledge and Information Systems 41 (2014): 647–665, 10.1007/s10115-013-0679-x.

[49] S. Zhang , J. He , X. Guo , et al., “Crystallization Dynamic Control of Perovskite Films With Suppressed Phase Transition and Reduced Defects for Highly Efficient and Stable All‐Inorganic Perovskite Solar Cells,” ACS Materials Letters 5 (2023): 1497–1505, 10.1021/acsmaterialslett.3c00275.

[50] Z. Iqbal , F. Zu , A. Musiienko , et al., “Interface Modification for Energy Level Alignment and Charge Extraction in CsPbI_3_ Perovskite Solar Cells,” ACS Energy Letters 8 (2023): 4304–4314, 10.1021/acsenergylett.3c01522. 37854052 PMC10580311

[51] X. Huang , C. Zhang , L. Cao , et al., “Enhancing Inverted Perovskite Solar Cells via Dipole‐Moment‐Tuned Self‐Assembled Monolayers With Efficiency of 25.75%,” Chemical Engineering Journal 511 (2025): 161967, 10.1016/j.cej.2025.161967.

[52] L. H. Hall , B. Mohney , and L. B. Kier , “The Electrotopological State: Structure Information at the Atomic Level for Molecular Graphs,” Journal of Chemical Information and Computer Sciences 31 (1991): 76–82, 10.1021/ci00001a012.

[53] X. Liu , X. Han , Y. Yu , D. He , J. Yi , and J. Chen , “Hydrogen Bond Network Suppresses Cascade Degradation for High‐Performance Air‐Processed Perovskite Solar Cells,” Chemical Engineering Journal 522 (2025): 167401, 10.1016/j.cej.2025.167401.

[54] F. Li , X. Deng , Z. Shi , et al., “Hydrogen‐Bond‐Bridged Intermediate for Perovskite Solar Cells With Enhanced Efficiency and Stability,” Nature Photonics 17 (2023): 478–484, 10.1038/s41566-023-01180-6.

[55] W. Zhang , Q.‐S. Li , and Z.‐S. Li , “Understanding the Surface Passivation Effects of Lewis Base in Perovskite Solar Cells,” Applied Surface Science 563 (2021): 150267, 10.1016/j.apsusc.2021.150267.

[56] A. K. Ramasamy , G. Rajamanickam , C. Anil Kumar , and C. Lung‐Chien , “Constructing a Hydrophobic Coating on MAPbI_3_ Perovskite Using Organic Molecule Based Phosphonium Iodide for Stable and Efficient Perovskite Solar Cells,” Journal of Materials Science: Materials in Electronics 36 (2025): 198, 10.1007/s10854-025-14240-0.

[57] X. Zeng , X. Ye , D. Liu , et al., “A New Simple and Efficient Molecular Descriptor for the Fast and Accurate Prediction of Log P,” Journal of Materials Informatics 5 (2025): 4, 10.20517/jmi.2024.61.

[58] A. U. Hassan and M. J. Aljaafreh , “Generating a Vast Chemical Space for High Polar Surface Area Triphenylamine Polymers by Machine Learning‐DFT Calculations Assisted Reverse Engineering for Photovoltaics,” Journal of Molecular Graphics and Modelling 139 (2025): 109078, 10.1016/j.jmgm.2025.109078. 40398132

[59] W. Gao , J. Ding , Q. Ma , et al., “Synergistic Modulation of Orientation and Steric Hindrance Induced by Alkyl Chain Length in Ammonium Salt Passivator Toward High‐Performance Inverted Perovskite Solar Cells and Modules,” Advanced Materials 37 (2025): 2413304, 10.1002/adma.202413304. 39551996

[60] J. Cai , J. Luo , S. Wang , and S. Yang , “Feature Selection in Machine Learning: A New Perspective,” Neurocomputing 300 (2018): 70–79, 10.1016/j.neucom.2017.11.077.

[61] Y. Ma , J. Gong , P. Zeng , and M. Liu , “Recent Progress in Interfacial Dipole Engineering for Perovskite Solar Cells,” Nano‐Micro Letters 15 (2023): 173, 10.1007/s40820-023-01131-4. 37420117 PMC10328907

[62] L. Hörmann , W. G. Stark , and R. J. Maurer , “Machine Learning and Data‐Driven Methods in Computational Surface and Interface Science,” npj Computational Materials 11 (2025): 196, 10.1038/s41524-025-01691-6. 40613091 PMC12213647

[63] A. Shrivastava , A. Gupta , and R. Girshick , “Training Region‐Based Object Detectors with Online Hard Example Mining,” paper presented at 2016 IEEE Conference on Computer Vision and Pattern Recognition (CVPR), Las Vegas, NV, USA, June 27–30, 2016, 10.1109/CVPR.2016.89.

[64] E. Unger and T. J. Jacobsson , “Tackling the Reproducibility Gap in Perovskite Research: A Vision for FAIR Data and Standardised Protocols,” EES Solar 2 (2026): 88–91, 10.1039/D5EL00163C.

[65] J. Yang , J. Wang , B. Shi , et al., “Improved Surface Hydrophobicity of Self‐Assembled Transport Layers Enables Perovskite/Silicon Tandem Solar Cells With Efficiency Approaching 31%,” Journal of Energy Chemistry 104 (2025): 749–755, 10.1016/j.jechem.2024.12.067.

[66] J. Jiang , B. Liu , L. Wang , et al., “Synergistic Interfacial Modification With Amphiphilic Astaxanthin for Efficient and Stable Perovskite Solar Cells,” Materials Science in Semiconductor Processing 204 (2026): 110278, 10.1016/j.mssp.2025.110278.

[67] S. Teale , M. Degani , B. Chen , E. H. Sargent , and G. Grancini , “Molecular Cation and Low‐Dimensional Perovskite Surface Passivation in Perovskite Solar Cells,” Nature Energy 9 (2024): 779–792, 10.1038/s41560-024-01529-3.

[68] Y. Miao , Y. Chen , H. Chen , X. Wang , and Y. Zhao , “Using Steric Hindrance to Manipulate and Stabilize Metal Halide Perovskites for Optoelectronics,” Chemical Science 12 (2021): 7231–7247, 10.1039/D1SC01171E. 34163817 PMC8171330

[69] Y.‐W. Yang , K. Xu , Z.‐N. Zhou , et al., “Organic Cation Conformational Flexibility Governs Mechanical Response in Organic–Inorganic Hybrid Materials,” Chemical Science 17 (2026): 1158–1166, 10.1039/D5SC06333G. 41283145 PMC12631573

[70] C. J. Bartel , C. Sutton , B. R. Goldsmith , et al., “New Tolerance Factor to Predict the Stability of Perovskite Oxides and Halides,” Science Advances 5 (2019): aav0693, 10.1126/sciadv.aav0693. PMC6368436 30783625

[71] L. Zhang , W. Wang , Y. Wei , et al., “Assessing the Effect of Excess PbI_2_ on the Photovoltaic Performance of CsPbI_3_ All‐Inorganic Perovskite Solar Cells,” Materials Today Communications 46 (2025): 112548, 10.1016/j.mtcomm.2025.112548.

[72] S. Li and J. T. S. Irvine , “Non‐Stoichiometry, Structure and Properties of Proton‐Conducting Perovskite Oxides,” Solid State Ionics 361 (2021): 115571, 10.1016/j.ssi.2021.115571.

[73] X. Qi , T. Zhang , F. Tan , et al., “Self‐Passivated Hybrid Perovskite Films for Improved Photovoltaic Performance of Solar Cells,” Journal of Materials Science 56 (2021): 6374–6384, 10.1007/s10853-020-05685-1.

[74] J. Fang , L. Wang , Z. Chen , et al., “Sulfonic Acid Functionalized Ionic Liquids for Defect Passivation via Molecular Interactions for High‐Quality Perovskite Films and Stable Solar Cells,” ACS Applied Materials & Interfaces 16 (2024): 23443–23451, 10.1021/acsami.4c04762. 38652094

[75] A. Gao , Y. Li , Y. He , et al., “Targeted Anchoring of all Cations With 5‐Bromopyridine‐3‐Sulfonic Acid for High‐Performance Perovskite Solar Cells,” ACS Applied Materials & Interfaces 17 (2025): 14129–14137, 10.1021/acsami.5c00581. 39994003

[76] K. Du , A. Wang , Y. Li , et al., “The Synergistic Effect of Phosphonic and Carboxyl Acid Groups for Efficient and Stable Perovskite Solar Cells,” Materials 16 (2023): 7306, 10.3390/ma16237306. 38068050 PMC10707214

[77] C. Ma , M.‐C. Kang , S.‐H. Lee , et al., “Facet‐Dependent Passivation for Efficient Perovskite Solar Cells,” Journal of the American Chemical Society 145 (2023): 24349–24357, 10.1021/jacs.3c09327. 37883799

[78] Z. Wu , E. Bi , L. K. Ono , et al., “Passivation Strategies for Enhancing Device Performance of Perovskite Solar Cells,” Nano Energy 115 (2023): 108731, 10.1016/j.nanoen.2023.108731.

[79] S. Wang , C. Yao , L. Li , et al., “Enhanced Passivation Durability in Perovskite Solar Cells via Concentration‐Independent Passivators,” Joule 8 (2024): 1105–1119, 10.1016/j.joule.2024.01.020.

[80] C. Tian , D. Liu , Y. Dong , et al., “Multifunctional Organic Molecule for Defect Passivation of Perovskite for High‐Performance Indoor Solar Cells,” Materials 18 (2025): 179, 10.3390/ma18010179. 39795824 PMC11722308

[81] C. Ma , C. Zhang , S. Chen , et al., “Interfacial Defect Passivation by Multiple‐Effect Molecule for Efficient and Stable Perovskite Solar Cells,” Solar Energy Materials and Solar Cells 262 (2023): 112499, 10.1016/j.solmat.2023.112499.

[82] W. Zhang , L. Chen , Z. Zou , et al., “Defect Passivation by a Multifunctional Phosphate Additive Toward Improvements of Efficiency and Stability of Perovskite Solar Cells,” ACS Applied Materials & Interfaces 14 (2022): 31911–31919, 10.1021/acsami.2c05956. 35796315

[83] C. A. Lipinski , F. Lombardo , B. W. Dominy , and P. J. Feeney , “Experimental and Computational Approaches to Estimate Solubility and Permeability in Drug Discovery and Development Settings,” Advanced Drug Delivery Reviews 64 (2012): 4–17, 10.1016/j.addr.2012.09.019. 11259830

[84] D. Rogers and M. Hahn , “Extended‐Connectivity Fingerprints,” Journal of Chemical Information and Modeling 50 (2010): 742–754, 10.1021/ci100050t. 20426451

[85] D. Butina , “Unsupervised Data Base Clustering Based on Daylight's Fingerprint and Tanimoto Similarity: A Fast and Automated Way to Cluster Small and Large Data Sets,” Journal of Chemical Information and Computer Sciences 39 (1999): 747–750, 10.1021/ci9803381.

[86] X.‐T. D. Tran , T.‐L. Phan , V.‐T. To , et al., “Integration of the Butina Algorithm and Ensemble Learning Strategies for the Advancement of a Pharmacophore Ligand‐Based Model: An In Silico Investigation of Apelin Agonists,” Frontiers in Chemistry 12 (2024): 1382319, 10.3389/fchem.2024.1382319. 38690013 PMC11058650

[87] L. J. P. van der Maaten and G. E. Hinton , “Visualizing High‐Dimensional Data Using T‐SNE,” Journal of Machine Learning Research 9 (2008): 2579–2605.

[88] V. N. Muppana , M. Samykano , M. M. Noor , H. Khir , Z. Said , and A. K. Pandey , “Integrated Machine Learning Framework for the Co‐optimization of Efficiency and Stability in Tin‐Based Perovskite Solar Cells,” Results in Engineering 29 (2026): 108767, 10.1016/j.rineng.2025.108767.

[89] L. Hirschfeld , K. Swanson , K. Yang , R. Barzilay , and C. W. Coley , “Uncertainty Quantification Using Neural Networks for Molecular Property Prediction,” Journal of Chemical Information and Modeling 60 (2020): 3770–3780, 10.1021/acs.jcim.0c00502. 32702986

[90] N. Meinshausen , “Quantile Regression Forests,” Journal of Machine Learning Research 7 (2006): 983–999.

[91] Z. Fang , B. Deng , Y. Jin , et al., “Surface Reconstruction of Wide‐Bandgap Perovskites Enables Efficient Perovskite/Silicon Tandem Solar Cells,” Nature Communications 15 (2024): 10554, 10.1038/s41467-024-54925-4. PMC11618607 39632852

[92] R. J. E. Westbrook , T. J. Macdonald , W. Xu , et al., “Lewis Base Passivation Mediates Charge Transfer at Perovskite Heterojunctions,” Journal of the American Chemical Society 143 (2021): 12230–12243, 10.1021/jacs.1c05122. 34342430

[93] Y. Zhu , L. Shi , H. Guo , et al., “Enhanced Luminescent Performance via Passivation of Surface Undercoordinated Pb Atoms in a CsPbBr_3_ Microplate,” Advanced Optical Materials 11 (2023): 2202428, 10.1002/adom.202202428.

