# Supplementary material for: Decoupling Intrinsic Molecular Efficacy From Platform Effects: An Interpretable Machine Learning Framework for Unbiased Perovskite Passivator Discovery
Source: Adv Sci (Weinh). 2026 Apr 30;13(41):e75486. doi: 10.1002/advs.75486 (PMC13335554; doi:10.1002/advs.75486)
Supplement: Supplementary file 1 — Supporting File: advs75486‐sup‐0001‐SuppMat.docx. [file ADVS-13-e75486-s001.docx]

*Supporting Information*

Decoupling Intrinsic Molecular Efficacy from Platform Effects: An Interpretable Machine Learning Framework for Unbiased Perovskite Passivator Discovery

Jing Zhang, Ziyuan Li, Shan Gao, Zhen Zhu, Jing Wang^*^, and Xiangmei Duan^*^

School of Physical Science and Technology, Ningbo University, Ningbo, China
E-mail: [wangjing4@nbu.edu.cn](mailto:wangjing4@nbu.edu.cn); duanxiangmei@nbu.edu.cn

**Note S1. Dataset Construction and Analysis**

To construct a high-quality, mechanism-homogeneous dataset for machine learning, we systematically curated literature on perovskite solar cells published between 2015 and 2025 from major publishers (Wiley, Elsevier, RSC, ACS, Nature Portfolio), applying stringent inclusion criteria.

First, to ensure chemical consistency, we focus exclusively on organic passivators, including small molecules, ionic compounds, or functionalized polymers. Purely inorganic materials (e.g., metal oxides) were excluded to avoid confounding mechanistic pathways. Second, to align with state-of-art device performance and minimize noise from suboptimal fabrication, only entries with an initial power conversion efficiency (I_PCE) > 18% were retained.

Critically, I_PCE was incorporated as a key input feature to enable decoupling of baseline device quality from the intrinsic molecular passivation efficacy. Moreover, rather than assuming ideal stoichiometry, we utilized actual precursor molar ratios (A:X, B:X)—a choice grounded in the understanding that precursor chemistry directly governs crystallization kinetics, defect density, and interfacial environment during passivation.

This curation yielded a database of 240 experimental entries encompassing 218 unique organic passivators (**Figure S1**). Each entry includes: (i) molecular structure; (ii) photovoltaic parameters (PCE, V_OC_, J_SC_, FF) for both control and passivated devices; and (iii) system-level descriptors (A-site composition, A:X, B:X). The full dataset is provided in **Table S1**.

Statistical analysis of photovoltaic outcomes conforms the dataset’s reliability and relevance (**Figure S2**): Passivation consistently enhances all key metrics. On average, PCE increases from 20.31% to 21.99% (+1.68% absolute), accompanied by gains in Voc (+0.04 V), Jsc (+0.4 mA cm^-2^), and FF (+3%). This systematic improvement validates the effectiveness of interface engineering within our dataset and establishes a robust foundation for predictive modeling.


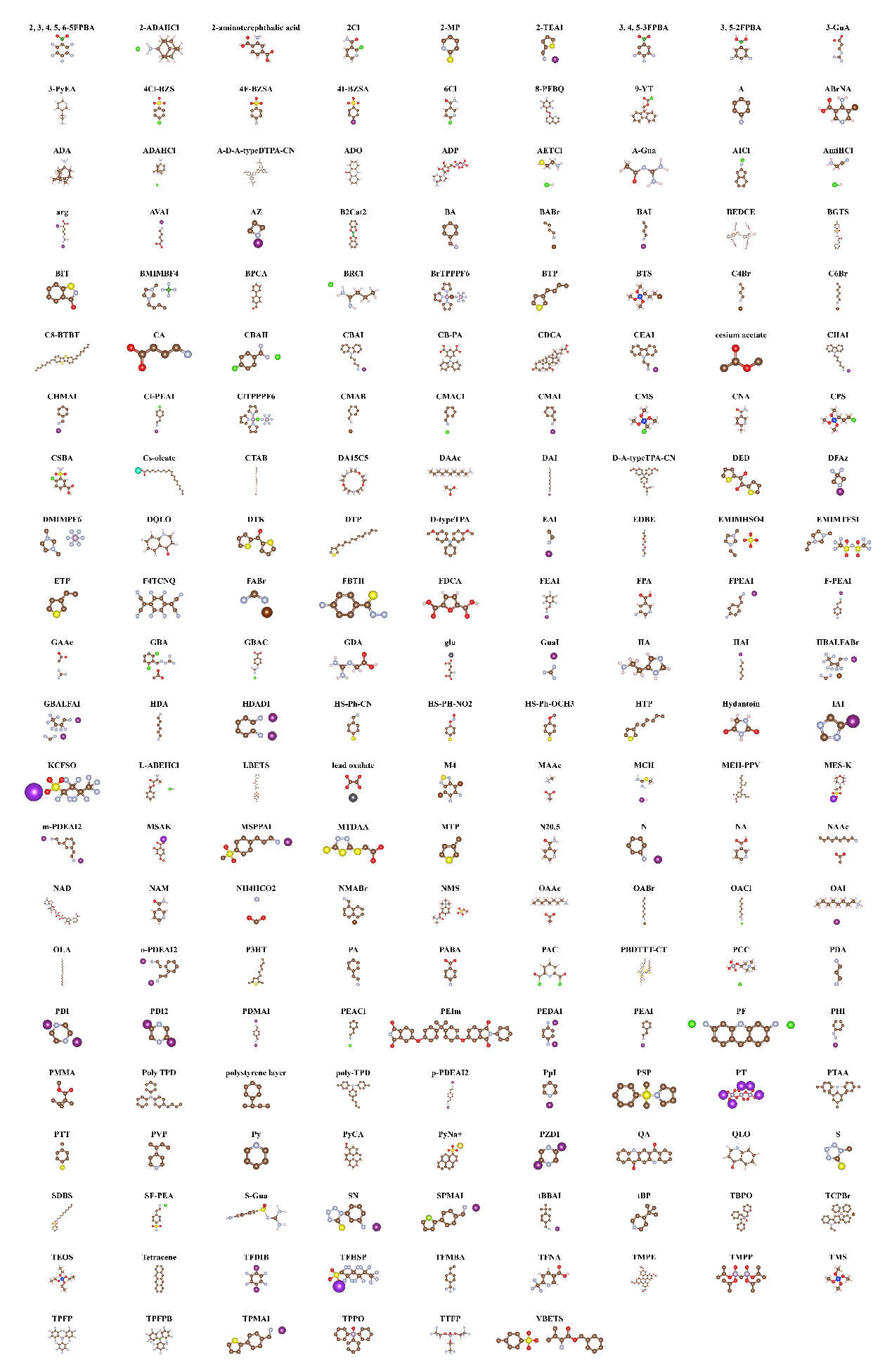


**Figure S1.** Chemical structures of the 218 unique passivators in the dataset. The collection encompasses a broad range of material classes, including organic small molecules, ionic compounds, and polymers. Each structure is displayed with its common name or abbreviation, arranged alphabetically for ease of reference.


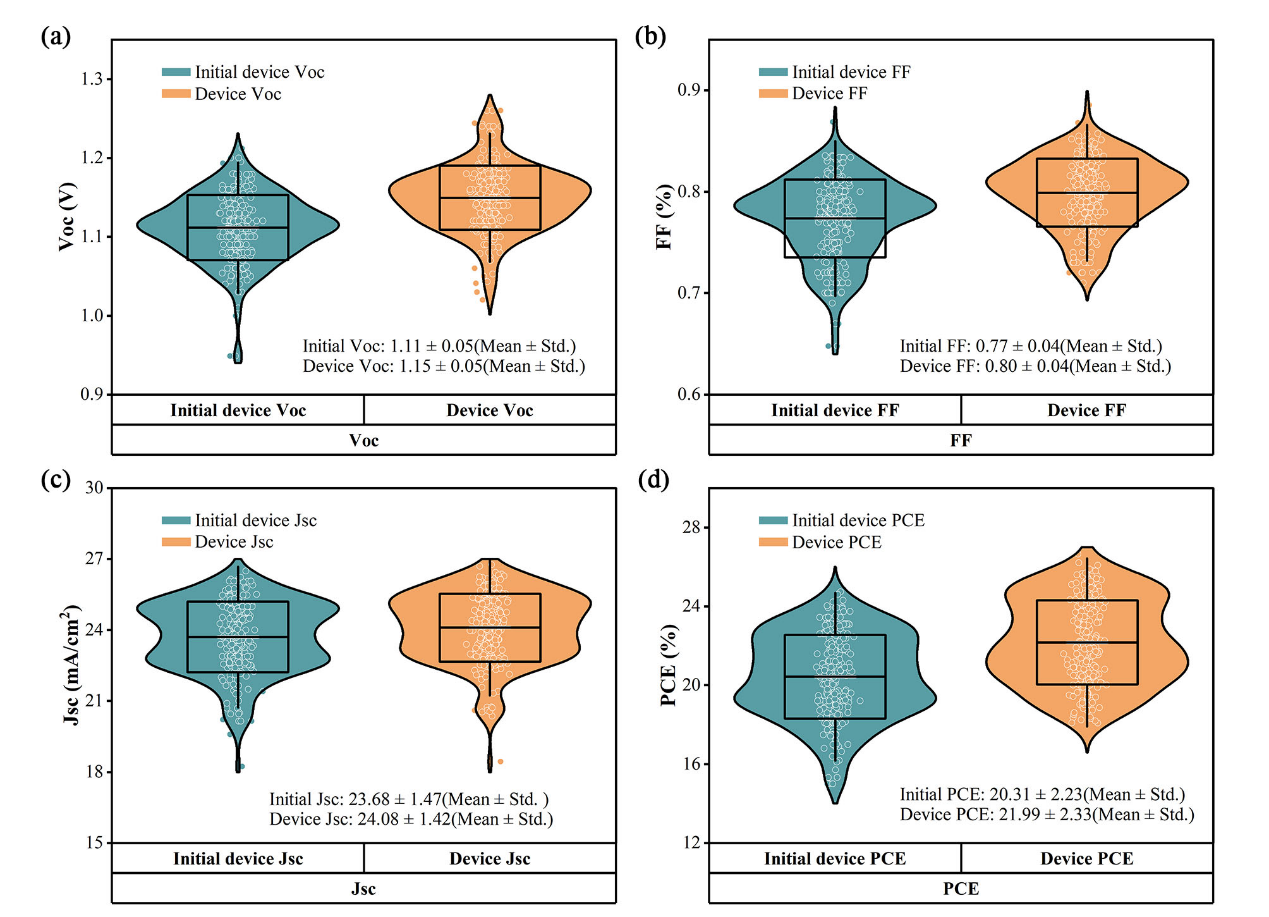


**Figure S2.** Statistical distribution of photovoltaic parameters for control and passivated devices. Violin plots comparing the distributions of key photovoltaic parameters between control (cyan) and passivated (orange) devices (N=240). Specifically, the plots show: a) Open-circuit voltage (V_OC_), b) Fill Factor (FF), c) Short-circuit current density (J_SC_), and d) Power conversion efficiency (PCE). Embedded box plots within each violin plot indicate median and interquartile range (IQR), with values representing mean±standard deviation.

**Table S1.** Curated dataset of 240 experimental entries for perovskite interface passivation. The dataset includes the following key fields: PCE of the passivated device, I_PCE of the corresponding control (unpassivated) device, Structure with chemical name or common abbreviation of the passivator, A:X / B:X (precursor molar ratios), rA (A-site cation composition), and DOI of the source publication.

| **PCE** | **I_PCE** | | **Structure** | | **A:X** | **B:X** | **rA** | **DOI** |
| --- | --- | --- | --- | --- | --- | --- | --- | --- |
| 21.06 | | 18.87 | | QA | 1:3 | 1:3 | MA | 10.1002/anie.202012095 |
| 18.51 | | 17.02 | | PEAI | 1:3 | 1:3 | Cs_0.05_MA_0.16_FA_0.79_ | 10.1002/adfm.201706923 |
| 19.13 | | 18.05 | | TMPP | 1:3 | 1:3 | Cs_0.05_FA_0.8_MA_0.15_ | 10.1002/adfm.201910710 |
| 21.04 | | 18.05 | | TPFP | 1:3 | 1:3 | Cs_0.05_FA_0.8_MA_0.15_ | 10.1002/adfm.201910710 |
| 19.1 | | 18.05 | | TPPO | 1:3 | 1:3 | Cs_0.05_FA_0.8_MA_0.15_ | 10.1002/adfm.201910710 |
| 21.47 | | 20.03 | | PDMAI | 0.563:3 | 1.218:3 | FA_0.75_MA_0.25_ | 10.1002/adfm.202002366 |
| 23.38 | | 20.61 | | OABr | 1.014:3 | 0.993:3 | Cs_0.063_6Rb_0.0273_FA_0.769_MA_0.14_ | 10.1002/adfm.202104251 |
| 23.62 | | 20.61 | | OACl | 1.014:3 | 0.993:3 | Cs_0.063_6Rb_0.0273_FA_0.769_MA_0.14_ | 10.1002/adfm.202104251 |
| 22.66 | | 20.61 | | OAI | 1.014:3 | 0.993:3 | Cs_0.063_6Rb_0.0273_FA_0.769_MA_0.14_ | 10.1002/adfm.202104251 |
| 21.41 | | 19.07 | | FBTH | 1.5:3 | 0.75:3 | H_0.5_Cs_0.5_ | 10.1002/adfm.202312638 |
| 25.11 | | 24.22 | | KCFSO | 1.1386:3 | 0.931:3 | FA_0.741_MA_0.259_ | 10.1002/adfm.202401945 |
| 25.14 | | 24.03 | | MES-K | 1.146:3 | 0.927:3 | F FA_0.741_MA_0.259_ | 10.1002/adfm.202404173 |
| 24.53 | | 22.05 | | FDCA | 1.095:3 | 0.952:3 | Cs_0.0435_FA_0.785_MA_0.1715_ | 10.1002/adfm.202404686 |
| 24.34 | | 22.53 | | PCC | 1.065:3 | 0.968:3 | Cs_0.043_FA_0.806_MA_0.151_ | 10.1002/adfm.202405556 |
| 23.77 | | 23.03 | | A-Gua | 1:3 | 1:3 | FA_0.85_Cs_0.15_ | 10.1002/adfm.202407897 |
| 24.34 | | 23.03 | | S-Gua | 1:3 | 1:3 | FA_0.85_Cs_0.15_ | 10.1002/adfm.202407897 |
| 25.47 | | 22.94 | | MSAK | 0.565:3 | 1.217:3 | FA_0.75_1MA_0.249_ | 10.1002/adfm.202409497 |
| 25.1 | | 23.5 | | M4 | 1.075:3 | 0.962:3 | Cs_0.0445_FA_0.785_MA_0.1705_ | 10.1002/adfm.202409852 |
| 23.75 | | 22.38 | | CMABr | 0.565:3 | 1.217:3 | FA_0.751_MA_0.249_ | 10.1002/adfm.202410878 |
| 25.47 | | 22.38 | | CMACl | 0.565:3 | 1.217:3 | FA_0.751_MA_0.249_ | 10.1002/adfm.202410878 |
| 24.37 | | 22.38 | | CMAI | 0.565:3 | 1.217:3 | FA_0.751_MA_0.249_ | 10.1002/adfm.202410878 |
| 25 | | 22.78 | | A-D-A-typeDTPA-CN | 0.568:3 | 1.216:3 | FA_0.746_MA_0.254_ | 10.1002/adfm.202412552 |
| 24.38 | | 22.78 | | D-A-typeTPA-CN | 0.568:3 | 1.216:3 | FA_0.746_MA_0.254_ | 10.1002/adfm.202412552 |
| 23.49 | | 22.78 | | D-typeTPA | 0.568:3 | 1.216:3 | FA_0.746_MA_0.254_ | 10.1002/adfm.202412552 |
| 19.9 | | 18.6 | | TMPE | 1:3 | 1:3 | MA | 10.1002/adfm.202413177 |
| 24.5 | | 23 | | TMPE | 0.968:3 | 1.016:3 | Cs^0.05^FA^0.85^MA^0.1^ | 10.1002/adfm.202413177 |
| 25.09 | | 23.08 | | PDI | 1:3 | 1:3 | Cs_0.15_FA_0.85_ | 10.1002/adfm.202419133 |
| 21.84 | | 23.08 | | PpI | 1:3 | 1:3 | Cs_0.15_FA_0.85_ | 10.1002/adfm.202419133 |
| 22.22 | | 19.99 | | 8-PFBQ | 0.992:3 | 1.004:3 | Cs_0.2_FA_0.8_ | 10.1002/adfm.202422175 |
| 25.54 | | 23.72 | | MSPPAI | 1.146:3 | 0.927:3 | Cs_0.036_FA_0.686_MA_0.278_ | 10.1002/adfm.202422266 |
| 19.2 | | 15.7 | | BA | 1:3 | 1:3 | MA | 10.1002/adma.201603062 |
| 19.57 | | 17.48 | | MMI | 1:3 | 1:3 | MA | 10.1002/adma.201800544 |
| 21.11 | | 19.61 | | B2Cat2 | 0.97:3 | 1.015:3 | Cs_0.0465_FA_0.8450_MA_0.1085_ | 10.1002/adma.201805085 |
| 21.9 | | 20.9 | | PTPD | 1:3 | 1:3 | Cs_0.0564_FA_0.9_MA_0.0 5_ | 10.1002/adma.201807435 |
| 21.7 | | 20.4 | | TBPO | 1.151:3 | 0.924:3 | Cs_0.0337_FA_0.6595_MA_0.3067_ | 10.1002/adma.201907396 |
| 21.2 | | 20.4 | | TPPO | 1.151:3 | 0.924:3 | Cs_0.0337_FA_0.6595_MA_0.3067_ | 10.1002/adma.201907396 |
| 22.7 | | 21.2 | | PEAI | 0.971:3 | 1.015:3 | Cs_0.05_FA_0.85_MA_0.1_ | 10.1002/adma.201907757 |
| 23.5 | | 21.2 | | tBBAI | 0.971:3 | 1.015:3 | Cs_0.05_FA_0.85_MA_0.1_ | 10.1002/adma.201907757 |
| 21.37 | | 19.36 | | Poly TPD | 1:3 | 1:3 | FA_0.85_MA_0.15_ | 10.1002/adma.202006087 |
| 21.15 | | 20.06 | | 2-TEAI | 1.003:3 | 0.998:3 | Cs_0.995_FA_0.005_ | 10.1002/adma.202007431 |
| 20.01 | | 17.75 | | EMIMHSO4 | 1.40:3 | 0.8:3 | H_0.555_Cs_0.445_ | 10.1002/adma.202106750 |
| 21.15 | | 19.12 | | DED) | 1.5:3 | 0.75:3 | H_0.5_Cs_0.5_ | 10.1002/adma.202210223 |
| 20.13 | | 19.12 | | DTK | 1.5:3 | 0.75:3 | H_0.5_Cs_0.5_ | 10.1002/adma.202210223 |
| 24.61 | | 21.3 | | PF | 1:3 | 1:3 | FA | 10.1002/adma.202308039 |
| 25.32 | | 23.75 | | CSBA | 0.613:3 | 1.194:3 | FA_0.679_MA_0.249_Rb_0.072_ | 10.1002/adma.202310710 |
| 25.165 | | 23.138 | | Hydantoin | 0.55:3 | 1.225:3 | FA_0.725_MA_0.275_ | 10.1002/adma.202313673 |
| 24.56 | | 22.86 | | TFHSP | 1:3 | 1:3 | Cs_0.05_FA_0.9215_MA_0.0285_ | 10.1002/adma.202407433 |
| 23.81 | | 21.82 | | DAAc | 1.036:3 | 0.982:3 | Cs_0.0435_FA_0.785_MA_0.1715_ | 10.1002/adma.202413304 |
| 25.34 | | 23.11 | | DAAc | 1.044:3 | 0.9778:3 | Cs_0.045_FA_0.864_MA_0.091_ | 10.1002/adma.202413304 |
| 22.84 | | 21.82 | | MAAc | 1.036:3 | 0.982:3 | Cs_0.0435_FA_0.785_MA_0.1715_ | 10.1002/adma.202413304 |
| 24.55 | | 23.11 | | MAAc | 1.044:3 | 0.9778:3 | Cs_0.045_FA_0.864_MA_0.091_ | 10.1002/adma.202413304 |
| 24.13 | | 21.82 | | NAAc | 1.036:3 | 0.982:3 | Cs_0.0435_FA_0.785_MA_0.1715_ | 10.1002/adma.202413304 |
| 25.79 | | 23.11 | | NAAc | 1.044:3 | 0.9778:3 | Cs_0.045_FA_0.864_MA_0.091_ | 10.1002/adma.202413304 |
| 23.34 | | 21.82 | | OAAc | 1.036:3 | 0.982:3 | Cs_0.0435_FA_0.785_MA_0.1715_ | 10.1002/adma.202413304 |
| 24.78 | | 23.11 | | OAAc | 1.044:3 | 0.9778:3 | Cs_0.045_FA_0.864_MA_0.091_ | 10.1002/adma.202413304 |
| 25.65 | | 23.97 | | 3-PyEA | 1.044:3 | 0.9778:3 | Cs_0.045_FA_0.8636_MA_0.0914_ | 10.1002/adma.202415100 |
| 25.27 | | 22.97 | | CB-PA | 1:3 | 1:3 | Cs_0.05_FA_0.95_ | 10.1002/adma.202500708 |
| 20.4 | | 16.8 | | PMMA | 1.001:3 | 0.999:3 | MA | 10.1002/admi.201701256 |
| 20.47 | | 19.01 | | PABA | 1:3 | 1:3 | MA | 10.1002/admi.201901584 |
| 18.24 | | 16.99 | | TEOS) | 1:3 | 1:3 | MA_0.7_FA_0.3_ | 10.1002/admi.201901716 |
| 24.51 | | 20.98 | | PT | 1.073:3 | 0.963:3 | Cs_0.0428_FA_0.731_MA_0.2262_ | 10.1002/advs.202404444 |
| 24.17 | | 20.92 | | 3-GuA | 0.625:3 | 1.188:3 | FA_0.663_MA_0.337_ | 10.1002/advs.202407401 |
| 19.44 | | 18.08 | | BTP | 0.981:3 | 1.009:3 | Cs_0.05_FA_0.81_MA_0.14_ | 10.1002/aenm.201703143 |
| 19.2 | | 18.08 | | DTP | 0.981:3 | 1.009:3 | Cs_0.05_FA_0.81_MA_0.14_ | 10.1002/aenm.201703143 |
| 19.17 | | 18.08 | | ETP | 0.981:3 | 1.009:3 | Cs_0.05_FA_0.81_MA_0.14_ | 10.1002/aenm.201703143 |
| 19.89 | | 18.08 | | HTP | 0.981:3 | 1.009:3 | Cs_0.05_FA_0.81_MA_0.14_ | 10.1002/aenm.201703143 |
| 18.82 | | 18.08 | | MTP | 0.981:3 | 1.009:3 | Cs_0.05_FA_0.81_MA_0.14_ | 10.1002/aenm.201703143 |
| 18.87 | | 18.08 | | TP | 0.981:3 | 1.009:3 | Cs_0.05_FA_0.81_MA_0.14_ | 10.1002/aenm.201703143 |
| 20.47 | | 19.32 | | AD | 1.006:3 | 0.997:3 | Cs_0.0497_FA_0.8077_MA_0.1425_ | 10.1002/aenm.201800275 |
| 20.93 | | 19.32 | | ADA | 1.006:3 | 0.997:3 | Cs_0.0497_FA_0.8077_MA_0.1425_ | 10.1002/aenm.201800275 |
| 20.8 | | 20.28 | | PMMA) | 1.014:3 | 0.993:3 | Cs_0.0636_Rb_0.0273_FA_0 .7692_MA_0.1399_ | 10.1002/aenm.201801208 |
| 20.54 | | 19.22 | | FPEAI | 0.9917:3 | 1.004:3 | Cs_0.1_FA_0.747_MA_0.153_ | 10.1002/aenm.201802595 |
| 20.28 | | 18.35 | | 2-MP | 0.9677:3 | 1.0161:3 | MA | 10.1002/aenm.201803573 |
| 18.74 | | 18.35 | | PTT | 0.9677:3 | 1.0161:3 | MA | 10.1002/aenm.201803573 |
| 19.02 | | 18.35 | | Py | 0.9677:3 | 1.0161:3 | MA | 10.1002/aenm.201803573 |
| 21.2 | | 20.52 | | ADAHCl | 1.013:3 | 0.994:3 | Cs_0.057_FA_0.802_MA_0.141_ | 10.1002/aenm.201803587 |
| 21.71 | | 21.23 | | BAI | 1.255:3 | 0.873:3 | FA_0.662_MA_0.338_ | 10.1002/aenm.201902740 |
| 21.89 | | 21.23 | | DAI | 1.255:3 | 0.873:3 | FA_0.662_MA_0.338_ | 10.1002/aenm.201902740 |
| 22.03 | | 21.23 | | OAI | 1.255:3 | 0.873:3 | FA_0.662_MA_0.338_ | 10.1002/aenm.201902740 |
| 23.1 | | 20.41 | | CHAI | 1:3 | 1:3 | FA | 10.1002/aenm.202102236 |
| 23.91 | | 20.41 | | CHMAI | 1:3 | 1:3 | FA | 10.1002/aenm.202102236 |
| 22.6 | | 20.1 | | EDBE | 0.945:3 | 1.027:3 | Cs_0.05_FA_0.81_MA_0.14_ | 10.1002/aenm.202102973 |
| 21.7 | | 20.1 | | HDA | 0.945:3 | 1.027:3 | Cs_0.05_FA_0.81_MA_0.14_ | 10.1002/aenm.202102973 |
| 24.33 | | 22.67 | | [EMIM]Br | 0.959:3 | 1.021:3 | FA | 10.1002/aenm.202103491 |
| 24.33 | | 22.21 | | CNA | 1:3 | 1:3 | FA_0.85_MA_0.15_ | 10.1002/aenm.202300610 |
| 23.99 | | 22.21 | | NA | 1:3 | 1:3 | FA_0.85_MA_0.15_ | 10.1002/aenm.202300610 |
| 24.57 | | 22.77 | | 2-ADAHCl | 0.825:3 | 1.087:3 | Cs_0.0658_GA_0.0234_MA_0.3058_FA_0.605_ | 10.1002/aenm.202400549 |
| 23.7 | | 21.78 | | CBAI | 1.207:3 | 0.896:3 | FA_0.735_MA_0.265_ | 10.1002/aenm.202401965 |
| 22.94 | | 21.78 | | CEAI | 1.207:3 | 0.896:3 | FA_0.735_MA_0.265_ | 10.1002/aenm.202401965 |
| 24.77 | | 21.78 | | CHAI | 1.207:3 | 0.896:3 | FA_0.735_MA_0.265_ | 10.1002/aenm.202401965 |
| 25.19 | | 21.78 | | OAI | 1.207:3 | 0.896:3 | FA_0.735_MA_0.265_ | 10.1002/aenm.202401965 |
| 24.05 | | 22.3 | | 3-PPA | 1:3 | 1:3 | Cs_0.05_FA_0.95_ | 10.1002/aenm.202402249 |
| 24.95 | | 22.95 | | NMS | 1:3 | 1:3 | Cs_0.1_FA_0.9_ | 10.1002/aenm.202402814 |
| 25.37 | | 22.97 | | FPA | 1:3 | 1:3 | Cs_0.05_FA_0.9025_MA_0.0475_ | 10.1002/aenm.202404335 |
| 25.9 | | 22.92 | | AmiHCl | 1.06:3 | 0.97:3 | Cs_0.035_FA_0.797_MA_0.168_ | 10.1002/aenm.202404638 |
| 24.82 | | 23.37 | | 9-YT | 0.968:3 | 1.016:3 | Cs_0.15_FA_0.85_ | 10.1002/aenm.202405571 |
| 25.6 | | 24.3 | | PAC | 1.0645:3 | 0.9677:3 | Cs_0.045_FA_0.82_MA_0.135_ | 10.1002/aenm.202406046 |
| 23.39 | | 22.09 | | ABrNA | 1:3 | 1:3 | Cs_0.1_FA_0.9_ | 10.1002/aenm.202500088 |
| 25.02 | | 23.89 | | ABrNA | 1:3 | 1:3 | Cs_0.05_FA_0.85_MA_0.1_ | 10.1002/aenm.202500088 |
| 22.46 | | 22.09 | | NA | 1:3 | 1:3 | Cs_0.1_FA_0.9_ | 10.1002/aenm.202500088 |
| 21.09 | | 19.13 | | 2-aminoterephthalic acid | 0.41:3 | 1.29:3 | MA | 10.1002/anie.201915422 |
| 23.25 | | 21.09 | | DMIMPF6 | 1:3 | 1:3 | Cs_0.08_FA_0.92_ | 10.1002/anie.202010987 |
| 19.9 | | 16.9 | | CMS | 0.997:3 | 1.001:3 | Cs_0.06_FA_0.79_MA_0.15_ | 10.1002/anie.202105512 |
| 19.5 | | 16.9 | | CPS | 0.997:3 | 1.001:3 | Cs_0.06_FA_0.79_MA_0.15_ | 10.1002/anie.202105512 |
| 20.8 | | 19.5 | | HA | 1.48:3 | 0.76:3 | Cs_0.513_H_0.487_ | 10.1002/anie.202109724 |
| 24.22 | | 22.6 | | GAAc | 1.147:3 | 0.926:3 | FA_0.741_MA_0.259_ | 10.1002/anie.202304568 |
| 24.7 | | 22.6 | | GDA | 1.147:3 | 0.926:3 | FA_0.741_MA_0.259_ | 10.1002/anie.202304568 |
| 20.86 | | 19.01 | | CA | 1.25:3 | 0.75:3 | Cs_0.5_MA_0.5_ | 10.1002/anie.202401751 |
| 21.86 | | 19.01 | | SA | 1.25:3 | 0.75:3 | Cs_0.5_MA_0.5_ | 10.1002/anie.202401751 |
| 24.08 | | 22.6 | | 3-APTCl | 1:3 | 1:3 | FA_0.85_MA_0.1_Cs_0.05_ | 10.1002/anie.202403610 |
| 25.01 | | 22.6 | | AETCl | 1:3 | 1:3 | FA_0.85_MA_0.1_Cs_0.05_ | 10.1002/anie.202403610 |
| 26.65 | | 24.74 | | MCH | 0.9:3 | 1.05:3 | Cs_0.555_FA_0.139_MA_0.306_ | 10.1002/anie.202419070 |
| 24.43 | | 23.08 | | BPCA | 1.144:3 | 0.928:3 | Cs_0.036_FA_0.688_MA_0.276_ | 10.1002/anie.202420369 |
| 24.87 | | 23.08 | | NACA | 1.144:3 | 0.928:3 | Cs_0.036_FA_0.688_MA_0.276_ | 10.1002/anie.202420369 |
| 25.67 | | 23.08 | | PyCA | 1.144:3 | 0.928:3 | Cs_0.036_FA_0.688_MA_0.276_ | 10.1002/anie.202420369 |
| 25.56 | | 22.03 | | L-ABEHCl | 1:3 | 1:3 | Cs_0.05_FA_0.95_ | 10.1002/anie.202425605 |
| 25.69 | | 22.46 | | TTFP | 1:3 | 1:3 | Cs_0.05_Rb_0.05_FA_0.85_MA_0.05_ | 10.1002/anie.202502244 |
| 21.02 | | 19.18 | | Az | 1.25:3 | 0.75:3 | Cs_0.5_MA_0.5_ | 10.1002/anie.202506282 |
| 22.05 | | 19.18 | | DFAz | 1.25:3 | 0.75:3 | Cs_0.5_MA_0.5_ | 10.1002/anie.202506282 |
| 22.09 | | 19.55 | | BrTPPPF6 | 1.2:3 | 0.9:3 | Cs_0.0367_Rb_0.015_FA_0.6977_MA_0.2506_ | 10.1002/eom2.12158 |
| 21.09 | | 19.55 | | ClTPPPF6 | 1.2:3 | 0.9:3 | Cs_0.0636_Rb_0.0273_FA_0.7692_MA_0.1399_ | 10.1002/eom2.12158 |
| 21.12 | | 19.32 | | 2Cl | 1:3 | 1:3 | MA | 10.1002/inf2.12379 |
| 23.13 | | 21.09 | | 2Cl | 1:3 | 1:3 | Cs_0.125_FA_0.875_ | 10.1002/inf2.12379 |
| 20.71 | | 19.32 | | 6Cl | 1:3 | 1:3 | MA | 10.1002/inf2.12379 |
| 22.51 | | 21.09 | | 6Cl | 1:3 | 1:3 | Cs_0.125_FA_0.875_ | 10.1002/inf2.12379 |
| 20 | | 19.32 | | N | 1:3 | 1:3 | MA | 10.1002/inf2.12379 |
| 22.39 | | 21.09 | | N | 1:3 | 1:3 | Cs_0.125_FA_0.875_ | 10.1002/inf2.12379 |
| 21.95 | | 19.76 | | CBAH | 1.124:3 | 0.938:3 | FA | 10.1002/smll.202104100 |
| 23.47 | | 21.06 | | PDI2 | 0.991:3 | 1.005:3 | Cs_0.05_FA_0.87_MA_0.08_ | 10.1002/smll.202208260 |
| 21.6 | | 19.55 | | TPFPB | 1:3 | 1:3 | FA_0.85_MA_0.15_ | 10.1002/solr.201900072 |
| 21.95 | | 19.47 | | CsAc | 1:3 | 1:3 | FA_0.85_MA_0.15_ | 10.1002/solr.201900220 |
| 18.39 | | 15.32 | | PEA | 1:3 | 1:3 | FA | 10.1002/solr.202000069 |
| 21.25 | | 15.32 | | SF-PEA | 1:3 | 1:3 | FA | 10.1002/solr.202000069 |
| 20.83 | | 19.45 | | PyNa+ | 0.978:3 | 1.011:3 | Cs_0.0517_FA_0.8621_MA_0.0862_ | 10.1002/solr.202100416 |
| 21.83 | | 19.91 | | BIT | 1:3 | 1:3 | Cs_0.05_FA_0.874_MA_0.076_ | 10.1002/solr.202100472 |
| 20.01 | | 17.49 | | Arg | 1.013:3 | 1.006:3 | Cs_0.123_FA_0.877_ | 10.1002/solr.202200858 |
| 19.35 | | 17.49 | | Glu | 1.013:3 | 1.006:3 | Cs_0.123_FA_0.877_ | 10.1002/solr.202200858 |
| 20.56 | | 19.25 | | TFMBA | 0.985:3 | 1.008:3 | Cs_0.0497_FA_0.8077_MA_0.1425_ | 10.1016/j.cej.2020.126712 |
| 18.35 | | 15 | | MEH-PPV | 1.8:3 | 0.6:3 | MA | 10.1016/j.jpowso ur.2017.12.082 |
| 19.01 | | 15 | | P3HT | 1.8:3 | 0.6:3 | MA | 10.1016/j.jpowso ur.2017.12.082 |
| 18.61 | | 15 | | PTAA | 1.8:3 | 0.6:3 | MA | 10.1016/j.jpowso ur.2017.12.082 |
| 20.56 | | 18.14 | | CDCA | 0.989:3 | 1.005:3 | Cs_0.0625_FA_0.7813_MA_0.1562_ | 10.1016/j.jpowsour.2020.228502 |
| 19.89 | | 18.61 | | ImI | 1:3 | 1:3 | MA | 10.1016/j.nanoen.2018.05.035 |
| 21.6 | | 18.9 | | ODAI2 | 0.947:3 | 1.026:3 | FA_0.8333_MA_0.1667_ | 10.1016/j.nanoen.2020.104892 |
| 20.04 | | 19.1 | | BMIMBF4 | 0.995:3 | 1.003:3 | Cs_0.0491_FA_0.7953_MA_0.1556_ | 10.1016/j.orgel.2020.105805 |
| 18.1 | | 16.1 | | PMMA | 1:3 | 1:3 | MA | 10.1021/acs.jpcc.6b12137 |
| 20.05 | | 18.03 | | AVAI | 1.095:3 | 0.952:3 | MA | 10.1021/acs.jpclett.0c02528 |
| 18.2 | | 17.5 | | BAI | 0.972:3 | 1.014:3 | Cs_0.0514_FA_0.7905_MA_0.1581_ | 10.1021/acsaem.0c00553 |
| 20.1 | | 17.5 | | F4TCNQ | 0.972:3 | 1.014:3 | Cs_0.0514_FA_0.7905_MA_0.1581_ | 10.1021/acsaem.0c00553 |
| 21.49 | | 18.72 | | PEACl | 1.286:3 | 0.857:3 | FA_0.1_MA_0.9_ | 10.1021/acsaem.1c02210 |
| 20.62 | | 17.43 | | PHI | 0.92:3 | 1.04:3 | Cs_0.05_FA_0.7885_MA_0.1615_ | 10.1021/acsami.0c10448 |
| 19.42 | | 18.41 | | SDBS | 1:3 | 1:3 | MA | 10.1021/acsami.0c14732 |
| 22.28 | | 20.49 | | ADP | 1.118:3 | 0.941:3 | Cs_0.126_FA_0.629_MA_0.245_ | 10.1021/acsami.4c01496 |
| 23.18 | | 20.49 | | NAD | 1.118:3 | 0.941:3 | Cs_0.126_FA_0.629_MA_0.245_ | 10.1021/acsami.4c01496 |
| 21.69 | | 20.49 | | NAM | 1.118:3 | 0.941:3 | Cs_0.126_FA_0.629_MA_0.245_ | 10.1021/acsami.4c01496 |
| 22.28 | | 21.87 | | 2,3,4,5,6-5FPBA | 0.982:3 | 1.009:3 | Cs_0.05_FA_0.807_MA_0.143_ | 10.1021/acsami.4c21627. |
| 22.76 | | 21.87 | | 3,4,5-3FPBA | 0.982:3 | 1.009:3 | Cs_0.05_FA_0.807_MA_0.143_ | 10.1021/acsami.4c21627. |
| 22.18 | | 21.87 | | 3,5-2FPBA | 0.982:3 | 1.009:3 | Cs_0.05_FA_0.807_MA_0.143_ | 10.1021/acsami.4c21627. |
| 22.18 | | 20.91 | | FPMAI | 0.975:3 | 1.012:3 | Cs_0.077_FA_0.806_MA_0.117_ | 10.1021/acsami.5c00985 |
| 21.65 | | 20.91 | | SPMAI | 0.975:3 | 1.012:3 | Cs_0.077_FA_0.806_MA_0.117_ | 10.1021/acsami.5c00985 |
| 23.15 | | 20.91 | | TPMAI | 0.975:3 | 1.012:3 | Cs_0.077_FA_0.806_MA_0.117_ | 10.1021/acsami.5c00985 |
| 22.73 | | 20.74 | | EMIMTFSI | 1:3 | 1:3 | Cs_0.065_FA_0.6545_MA_0.2805_ | 10.1021/acsami.5c01012 |
| 23.6 | | 19.39 | | BTS | 1.116:3 | 0.942:3 | FA_0.843_MA_0.157_ | 10.1021/acsami.5c01522. |
| 24.75 | | 22.71 | | DA15C5 | 1：3 | 1:3 | Cs_0.048_FA_0.873_MA_0.079_ | 10.1021/acsami.5c02442 |
| 20.46 | | 19.24 | | polystyrene layer | 0.9375:3 | 1.031:3 | Cs_0.05_FA_0.7885_MA_0.1615_ | 10.1021/acsami.8b04776 |
| 20.9 | | 19.7 | | cesium acetate | 1:3 | 1:3 | Cs_0.05_FA_0.8_MA_0.15_ | 10.1021/acsami.8b10616 |
| 18.31 | | 17.65 | | Cs-oleate | 1.033:3 | 0.984:3 | Cs_0.0476_FA_0.7905_MA_0.1619_ | 10.1021/acsami.9b08026 |
| 20.18 | | 18.69 | | DMEDAI2 | 1:3 | 1:3 | MA | 10.1021/acsami.9b17851 |
| 19.43 | | 18.83 | | BAI | 0.975:3 | 1.012:3 | Cs_0.0476_FA_0.8095_MA_0.1429_ | 10.1021/acsami.9b17930 |
| 20.62 | | 18.83 | | HAI | 0.975:3 | 1.012:3 | Cs_0.0476_FA_0.8095_MA_0.1429_ | 10.1021/acsami.9b17930 |
| 21.53 | | 19.48 | | OLA | 1.194:3 | 0.903:3 | Cs_0.0378_FA_0.6280_MA_0.3342_ | 10.1021/acsenergylett.0c00279 |
| 22.78 | | 20.1 | | HBAI.FABr | 0.977:3 | 1.012:3 | Cs_0.05_FA_0.85_MA_0.1_ | 10.1021/acsenergylett.0c01664 |
| 21.92 | | 20.1 | | HBAI.FACl | 0.977:3 | 1.012:3 | Cs_0.05_FA_0.85_MA_0.1_ | 10.1021/acsenergylett.0c01664 |
| 22.25 | | 20.1 | | HBAI.FAI | 0.977:3 | 1.012:3 | Cs_0.05_FA_0.85_MA_0.1_ | 10.1021/acsenergylett.0c01664 |
| 21.29 | | 19.71 | | MTDAA | 1.201:3 | 0.899:3 | Cs_0.037_Rb_0.015_FA_0.697_MA_0.251_ | 10.1021/acsenergylett.1c00794 |
| 21.94 | | 19.17 | | C8-BTBT | 0.981:3 | 1.009:3 | Cs_0.05_FA_0.85_MA_0.1_ | 10.1021/acsenergylett.1c01898 |
| 23.04 | | 22.11 | | NMABr | 0.563:3 | 1.218:3 | Cs_0.0215_FA_0.751_MA_0.2245_ | 10.1021/acsmaterialslett.4c02099 |
| 22.62 | | 21.67 | | ADO | 0.583:3 | 1.208:3 | Cs_0.09_FA_0.695_MA_0.215_ | 10.1021/acsmaterialslett.5c00057 |
| 20.71 | | 21.67 | | DQLO | 0.583:3 | 1.208:3 | Cs_0.09_FA_0.695_MA_0.215_ | 10.1021/acsmaterialslett.5c00057 |
| 23.05 | | 21.67 | | QLO | 0.583:3 | 1.208:3 | Cs_0.09_FA_0.695_MA_0.215_ | 10.1021/acsmaterialslett.5c00057 |
| 20.9 | | 19.8 | | PVP | 0.964:3 | 1.018:3 | Cs_0.04_FA_0.8_MA_0.16_ | 10.1021/acsomega.8b00555 |
| 21.98 | | 20.76 | | PDI | 1.182:3 | 0.91:3 | Cs_0.038_FA_0.694_MA_0.268_ | 10.1021/jacs.0c09845 |
| 23.1 | | 20.76 | | PI | 1.182:3 | 0.91:3 | Cs_0.038_FA_0.694_MA_0.268_ | 10.1021/jacs.0c09845 |
| 20.5 | | 20.45 | | TFDIB | 0.971:3 | 1:3 | Cs_0.05_FA_0.855_MA_0.095_ | 10.1021/jacs.9b13701 |
| 20.5 | | 19.2 | | N | 1:3 | 1.05:3 | Cs_0.1_FA_0.9_ | 10.1038/s41467 018-06709-w |
| 20.5 | | 19.2 | | S | 1:3 | 1.05:3 | Cs_0.1_FA_0.9_ | 10.1038/s41467 018-06709-w |
| 20.9 | | 19.2 | | SN | 1:3 | 1.05:3 | Cs_0.1_FA_0.9_ | 10.1038/s41467 018-06709-w |
| 22.3 | | 20.72 | | EAI | 0.98:3 | 1.0098:3 | Cs_0.07_FA_0.9_MA_0.03_ | 10.1038/s41467-019-10985-5 |
| 20.9 | | 20.72 | | GuaI | 0.98:3 | 1.0098:3 | Cs_0.07_FA_0.9_MA_0.03_ | 10.1038/s41467-019-10985-5 |
| 21.6 | | 20.72 | | IAI | 0.98:3 | 1.0098:3 | Cs_0.07_FA_0.9_MA_0.03_ | 10.1038/s41467-019-10985-5 |
| 19.63 | | 21.94 | | m-PDEAI2 | 1.064:3 | 0.968:3 | Cs_0.045_FA_0.736_MA_0.219_ | 10.1038/s41467-021-26754-2 |
| 23.92 | | 21.94 | | o-PDEAI2 | 1.064:3 | 0.968:3 | Cs_0.045_FA_0.736_MA_0.219_ | 10.1038/s41467-021-26754-2 |
| 21.09 | | 21.94 | | p-PDEAI2 | 1.064:3 | 0.968:3 | Cs_0.045_FA_0.736_MA_0.219_ | 10.1038/s41467-021-26754-2 |
| 25.39 | | 23.43 | | lead oxalate | 1.154:3 | 0.923:3 | FA | 10.1038/s41467-023-41853-y |
| 19.21 | | 19.65 | | PEDAI | 1:3 | 1:3 | Cs_0.12_Rb_0.04_FA_0.84_ | 10.1038/s41467-024-45228-9 |
| 23.17 | | 19.65 | | PZDI | 1:3 | 1:3 | Cs_0.12_Rb_0.04_FA_0.84_ | 10.1038/s41467-024-45228-9 |
| 23.82 | | 23.44 | | BGTS | 1.029：3 | 0.985：3 | Cs_0.044_FA_0.79_MA_0.166_ | 10.1038/s41467-025-56068-6 |
| 24.77 | | 23.44 | | LBETS | 1.029：3 | 0.985：3 | Cs_0.044_FA_0.79_MA_0.166_ | 10.1038/s41467-025-56068-6 |
| 25.26 | | 23.44 | | VBETS | 1.029：3 | 0.985：3 | Cs_0.044_FA_0.79_MA_0.166_ | 10.1038/s41467-025-56068-6 |
| 20.03 | | 18.74 | | AICl | 0.967:3 | 1.016:3 | MA | 10.1038/s41560-023-01295-8 |
| 24.93 | | 22.64 | | AICl | 1:3 | 1:3 | Cs_0.05_FA_0.95_ | 10.1038/s41560-023-01295-8 |
| 22.4 | | 20.03 | | AICl) | 1:3 | 1:3 | Cs_0.05_FA_0.8075_MA_0.1425_ | 10.1038/s41560-023-01295-8 |
| 24.85 | | 22.89 | | GBA | 1:3 | 1:3 | FA_0.25_MA_0.75_ | 10.1038/s41560-023-01358-w |
| 18.08 | | 16.18 | | BABr | 0.996:3 | 1.002:3 | Cs_0.2_FA_0.8_ | 10.1038/s41566-021-00829-4 |
| 20.67 | | 18.58 | | BABr | 1:3 | 1:3 | MA_0.7_FA_0.3_ | 10.1038/s41566-021-00829-4 |
| 21.54 | | 19.61 | | BABr | 0.995:3 | 1.002:3 | Rb_0.047_Cs_0.04_FA_0.763_MA_0.15_ | 10.1038/s41566-021-00829-4 |
| 23.78 | | 21.71 | | BABr | 1.156:3 | 0.922:3 | Cs_0.024_FA_0.74_MA_0.236_ | 10.1038/s41566-021-00829-4 |
| 24.8 | | 22.8 | | GBAC | 1.064:3 | 0.968:3 | Cs_0.054_FA_0.726_MA_0.22_ | 10.1038/s41566-023-01180-6 |
| 26.09 | | 24.61 | | PSP | 1.132:3 | 0.934:3 | Cs_0.04_FA_0.73_MA_0.23_ | 10.1038/s41586-023-06784-0 |
| 24.62 | | 23.45 | | NH4HCO2 | 0.583:3 | 1.21:3 | Cs_0.215_FA_0.556_MA_0.229_ | 10.1038/s43246-024-00673-3 |
| 20.5 | | 18.9 | | FABr | 0.975:3 | 1.0125:3 | FA_0.846_MA_0.154_ | 10.1039/C6EE03182J |
| 19.1 | | 19 | | HS Ph-SCH3 | 0.972:3 | 1.014:3 | Cs_0.0514_FA_0.79_MA_0.1586_ | 10.1039/C8EE00754C |
| 19.6 | | 18.5 | | HS-Ph-CN | 0.972:3 | 1.014:3 | Cs_0.0514_FA_0.79_MA_0.1586_ | 10.1039/C8EE00754C |
| 20 | | 19 | | HS-PH-NO2 | 0.972:3 | 1.014:3 | Cs_0.0514_FA_0.79_MA_0.1586_ | 10.1039/C8EE00754C |
| 19.4 | | 19 | | HS-Ph-OCH3 | 0.972:3 | 1.014:3 | Cs_0.0514_FA_0.79_MA_0.1586_ | 10.1039/C8EE00754C |
| 19.02 | | 17.2 | | BEDCE | 1.005:3 | 0.997:3 | MA | 10.1039/C8TA09724K |
| 22 | | 20.7 | | C4Br | 1.139886:3 | 0.93:3 | FA_0.696_MA_0.304_ | 10.1039/C9EE00751B |
| 22.4 | | 20.7 | | C6Br | 1.139886:3 | 0.93:3 | FA_0.696_MA_0.304_ | 10.1039/C9EE00751B |
| 22.1 | | 20.7 | | C8Br | 1.139886:3 | 0.93:3 | FA_0.696_MA_0.304_ | 10.1039/C9EE00751B |
| 19.2 | | 16.4 | | tBP | 1:3 | 1:3 | FA_0.85_MA_0.15_ | 10.1039/C9EE01773A |
| 18.95 | | 16.99 | | CTAB | 1:3 | 1:3 | MA | 10.1039/C9TA02631B |
| 20.28 | | 19.62 | | CTAB | 1:3 | 1:3 | FA_0.95_MA_0.05_ | 10.1039/C9TA02631B |
| 20.13 | | 18.52 | | TCPBr | 1:3 | 1:3 | MA | 10.1039/C9TA12597C |
| 19.41 | | 18.52 | | TCPI | 1:3 | 1:3 | MA | 10.1039/C9TA12597C |
| 21.19 | | 19.52 | | EPC | 1.2:3 | 0.9:3 | FA_0.716_MA_0.284_ | 10.1039/D0TA02222E |
| 20.31 | | 19.22 | | HDADI | 0.975:3 | 1.012:3 | Cs_0.0477_FA_0.8095_MA_0.1428_ | 10.1039/D0TA02437F |
| 21.01 | | 19.45 | | PEIm | 1:3 | 1:3 | Cs_0.091_FA_0.795_MA_0.141_ | 10.1039/D0TA05496H |
| 18.7 | | 17.9 | | BAI | 1.026:3 | 0.987:3 | MA | 10.1039/D1RA02260A |
| 23.27 | | 21.59 | | BRCl | 0.641:3 | 1.179:3 | Cs_0.165_FA_0.665_MA_0.17_ | 10.1039/D1TA08092J |
| 20.45 | | 16.64 | | TFNA | 0.91:3 | 1.045:3 | Cs_0.026_FA_0.813_MA_0.161_ | 10.1039/d4tc02432j |
| 21.6 | | 19.3 | | Tetracene | 1.013:3 | 1.021:3 | Cs_0.06_FA_0.7935_MA_0.1501_ | 10.1126/sciadv.aav2012 |
| 22.16 | | 20.62 | | FEAI | 0.958:3 | 1.021:3 | Cs_0.04_FA_0.9201_MA_0.0398_ | 10.1126/sciadv.aaw2543 |
| 23.3 | | 21.8 | | BHC | 1:3 | 1:3 | MA_0.6_FA_0.4_ | 10.1126/sciadv.abe8130 |
| 23.4 | | 19.2 | | BHC | 1:3 | 1:3 | MA_0.7_FA_0.3_ | 10.1126/sciadv.abe8130 |
| 23.07 | | 21.57 | | Cl-PEAI | 0.993:3 | 1.003:3 | Cs_0.05_FA_0.792_MA_0.158_ | 10.1126/sciadv.abj7930 |
| 23.72 | | 21.57 | | F-PEAI | 0.993:3 | 1.003:3 | Cs_0.05_FA_0.792_MA_0.158_ | 10.1126/sciadv.abj7930 |
| 22.04 | | 21.57 | | PEAI | 0.993:3 | 1.003:3 | Cs_0.05_FA_0.792_MA_0.158_ | 10.1126/sciadv.abj7930 |
| 26.22 | | 23.93 | | 4Cl-BZS | 1:3 | 1:3 | Cs_0.05_FA_0.85_MA_0.1_ | 10.1126/science.adm9474 |
| 25.81 | | 23.93 | | 4F-BZSA | 1:3 | 1:3 | Cs_0.05_FA_0.85_MA_0.1_ | 10.1126/science.adm9474 |
| 24.65 | | 23.93 | | 4I-BZSA | 1:3 | 1:3 | Cs_0.05_FA_0.85_MA_0.1_ | 10.1126/science.adm9474 |

**Note S2. Machine Learning Modeling**

To comprehensively and objectively evaluate the predictive performance of each machine learning model, we employed three standard regression metrics: the coefficient of determination (R^2^), Mean Absolute Error (MAE), and Root Mean Square Error (RMSE). Their calculation formulas are given as follows:

$$\boldsymbol{R}^{\boldsymbol{2}}\boldsymbol{=}\frac{\left[ \sum_{\boldsymbol{i=1}}^{\boldsymbol{n}} \left( \boldsymbol{x}_{\boldsymbol{i}}\boldsymbol{-}\hat{\boldsymbol{x}} \right)\left( \boldsymbol{x}_{\boldsymbol{i}}^{\boldsymbol{'}}\boldsymbol{-}{\hat{\boldsymbol{x}}}^{\boldsymbol{'}} \right) \right]}{\sum_{\boldsymbol{i=1}}^{\boldsymbol{n}} \left( \boldsymbol{x}_{\boldsymbol{i}}\boldsymbol{-}\hat{\boldsymbol{x}} \right)^{\boldsymbol{2}}\boldsymbol{\cdot}\sum_{\boldsymbol{i=1}}^{\boldsymbol{n}} \left( \boldsymbol{x}_{\boldsymbol{i}}^{\boldsymbol{'}}\boldsymbol{-}{\hat{\boldsymbol{x}}}^{\boldsymbol{'}} \right)^{\boldsymbol{2}}}$$

$$\boldsymbol{MAE=}\frac{\sum_{\boldsymbol{i=1}}^{\boldsymbol{n}} \left| \boldsymbol{x}_{\boldsymbol{i}}^{\boldsymbol{'}}\boldsymbol{-x} \right|}{\boldsymbol{n}}$$

$$\boldsymbol{RMSE}\boldsymbol{=}\sqrt{\frac{\sum_{\boldsymbol{i=1}}^{\boldsymbol{n}} \left( \boldsymbol{x}_{\boldsymbol{i}}^{\boldsymbol{'}}\boldsymbol{-}\boldsymbol{x}_{\boldsymbol{i}} \right)^{\boldsymbol{2}}}{\boldsymbol{n}}}$$

where $\boldsymbol{n}$ is the total number of data points, $\boldsymbol{x}_{\boldsymbol{i}}$ and$\boldsymbol{x}_{\boldsymbol{i}}^{\boldsymbol{'}}$represent the true and predicted values, respectively, and $\hat{\boldsymbol{x}}$ and ${\hat{\boldsymbol{x}}}^{\boldsymbol{'}}$ denote the means of the true and predicted values.

To ensure relevance and minimize redundancy, we curated an initial pool of candidate features encompassing system parameters, molecular descriptors, and baseline device performance. A multi-criteria feature selection strategy—integrating SHAP analysis, Random Forest feature importance (based on MSE reduction), and Pearson correlation—was then applied to identify the final 21-dimensional feature set (Table 1). This process ensures that each selected feature contributes unique information, thereby reducing multicollinearity and mitigating overfitting risks associated with high dimensional inputs.

All calculations were implemented in Python using Pandas and Scikit-learn, with input features standardized to zero mean and unit variance. We adopted a rigorous and reproducible protocol for model training and evaluation. The dataset was first partitioned into a training set (80%) and an isolated hold-out test set (20%). To assess robustness against data splitting variability, this random split was repeated 10 times independently; all reported performance metrics represent the mean standard deviation across these replicates.

For each six regression algorithms, hyperparameters were optimized via 10-fold cross-validation on the training subset. To improve model robustness—particularly for challenging or atypical cases—we incorporated an iterative re-weighting strategy inspired by Hard Example Mining.^[1]^ Within each cross-validation fold, a preliminary model identified "hard samples" as the top 10% of instances exhibiting the largest prediction errors on the validation subset. These samples were then assigned higher loss weights during retraining, effectively steering the learning process toward difficult examples. Crucially, the original train/validation partition was strictly preserved throughout this procedure to eliminate any risk of data leakage. Following hyperparameters optimization (**Table S2**), Final models were retrained on the entire 80% training set and evaluated on the held-out test set to report unbiased generalization performance.

**Table S2.** Optimized hyperparameter configurations for the six regression models. Hyperparameters were tuned via 10-fold cross-validation with hard sample mining to enhance model robustness and generalization.

| Algorithm | Hyperparameters |
| --- | --- |
| Random Forest (RF) | n_estimators: 100, max_depth: 20, max_features: 0.7, min_samples_leaf: 2, min_samples_split: 5 |
| XGBoost | n_estimators: 100, learning_rate: 0.05, max_depth: 3, subsample: 0.8, colsample_bytree: 0.8 |
| LightGBM | n_estimators: 100, learning_rate: 0.05, num_leaves: 31, colsample_bytree: 0.8 |
| Support Vector Machine (SVM) | kernel: 'rbf', C: 10, gamma: 'scale', epsilon: 0.5 |
| K-Nearest Neighbors (KNN) | n_neighbors: 5, weights: 'distance', p: 2 |
| Neural Network (NN) | hidden_layer_sizes: (50, 25), activation: 'tanh', alpha: 0.01, learning_rate_init: 0.01 |

**Note S3. Validation and Comparative Analysis of Feature Sets**

To rigorously validate the effectiveness of our feature engineering, we conducted controlled benchmarks using a standardized ML workflow (Random Forest, without hard sample mining) across four scenarios: (i) our full 21D feature set, (ii) a reduced 15D subset, and (iii-iv) two literature-based baselines from Liu et al.^[2]^ and Zhi et al.^[3]^

Quantitative results (**Table S3**, **Figure S3**) demonstrate that models trained on our feature sets (both 21D and 15D) achieve consistently superior generalization, with test R^2^≈0.826. In contrast, both literature baselines exhibit signs of overfitting. The descriptor set from Zhi et al.—based primarily on atomic counts—suffers from significant overfitting (train R^2^=0.971vs test R^2^=0.797), likely because it fails to encode directional interactions critical for passivation. Liu et al.'s set, which incorporates E-state indices to capture hydrogen-bonding propensity, performs better (test R^2^=0.804) but lacks descriptors for electronic properties such as molecular dipole moments and charge distribution.

The advantage of our feature set arises from its multidimensional design, which holistically integrates system-level parameters, hydrogen-bonding capacity, and fine-grained electronic descriptors (e.g., dipole moments, Gasteiger partial charges). This "bonding+ electronics" representation enables the model to more accurately decode the physical origins of passivation efficacy. Notably, the near-equivalent performance of the 15-feature subset underscores the high information density and efficiency of our engineered descriptors—demonstrating that redundancy is minimal and each feature contributes meaningfully to predictive power.


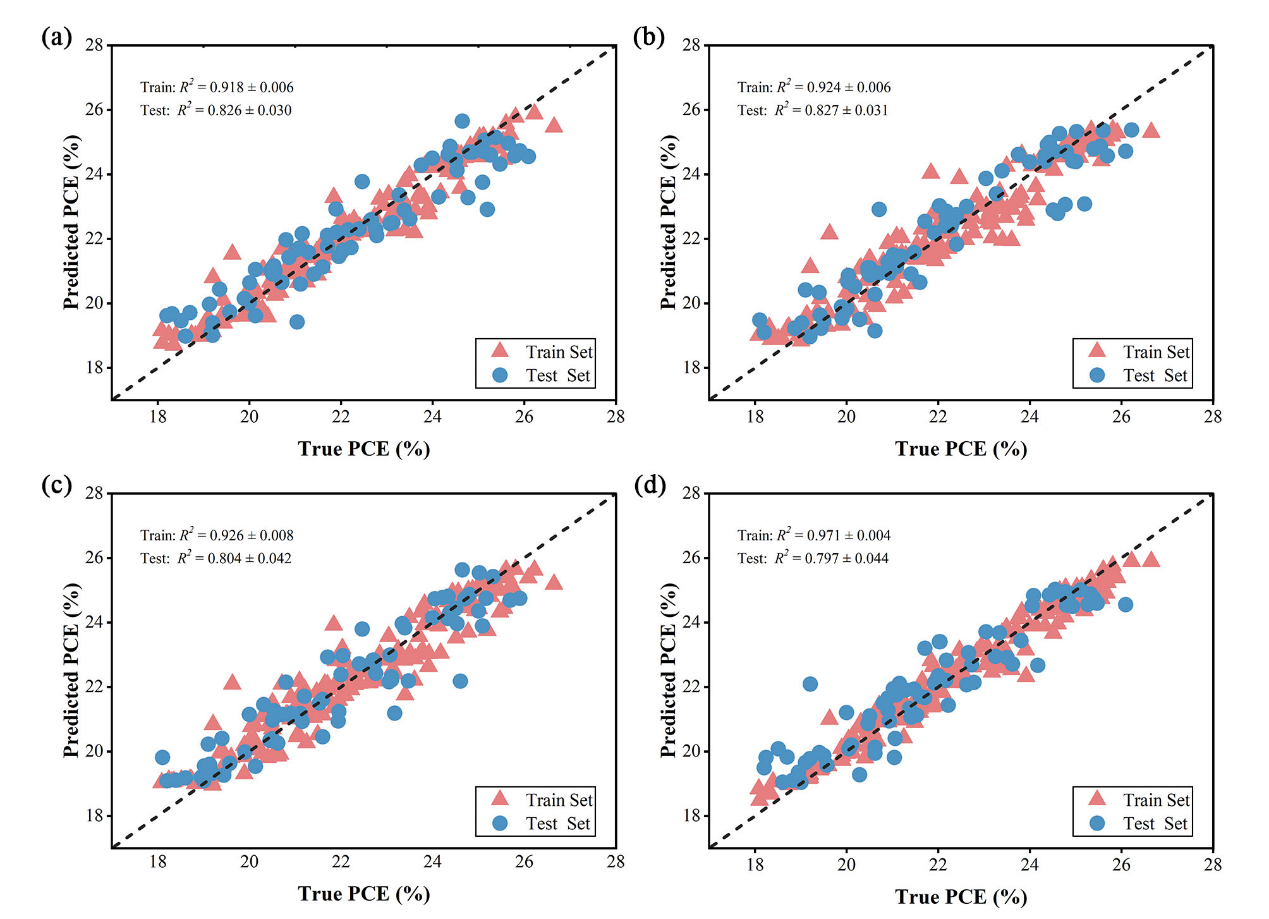


**Figure S3.** Performance comparison of RF models using different feature sets. Parity plots comparing the performance of RF models trained on different feature sets: a) Full 21-feature set, b) Top-15 feature subset, c) Feature set from Liu et al., and d) Feature set from Zhi et al.. Data points represent predictions for the training set (red triangles) and test set (blue circles). The insets display the mean R^2^±standard deviation across 10 independent runs, providing a robust evaluation of model stability and predictive accuracy.

**Table S3.** Quantitative performance metrics for RF models trained on different feature sets. Reported values are mean±standard deviation across 10 independent runs. Both MAE and RMSE are expressed in percentage points (% PCE).

| Model | Train-R^2^ | Test-R^2^ | Train-MAE | Test-MAE | Train-RMSE | Test-RMSE |
| --- | --- | --- | --- | --- | --- | --- |
| This Work  (21 features) | 0.918 ± 0.006 | 0.826 ± 0.030 | 0.449 ± 0.013 | 0.698 ± 0.053 | 0.605 ± 0.020 | 0.901 ± 0.079 |
| This Work  (15 features) | 0.924 ± 0.006 | 0.826 ± 0.034 | 0.448 ± 0.022 | 0.700 ± 0.053 | 0.601 ± 0.028 | 0.912 ± 0.070 |
| Liu et al. | 0.926 ± 0.008 | 0.804 ± 0.042 | 0.433 ± 0.023 | 0.711 ± 0.072 | 0.578 ± 0.033 | 0.934 ± 0.099 |
| Zhi et al. | 0.971 ± 0.004 | 0.797 ± 0.044 | 0.276 ± 0.019 | 0.737 ± 0.073 | 0.360 ± 0.025 | 0.955 ± 0.107 |

**Note S4. Supplementary Analysis and Discussion of Model Performance**

We further examined the performance disparities among the six ML algorithms and presented a detailed residual analysis of the optimal Random Forest (RF) model. Quantitative metrics and prediction scatter plots are provided in **Table S4** and **Figure S4**.

Tree-based ensemble models—RF, XGBoost, and LightGBM—consistently outperformed others, reflecting their strength in handling tabular data with heterogenerous features. Among them, RF achieved the best trade-off between generalization and robustness, delivering a test R^2^=0.914. While XGBoost (R^2^=0.893) and LightGBM (R^2^=0.885) showed competitive performance, both exhibited a modest increase in overfitting—likely attributable to their boosting mechanisms frameworks, which can overemphasize noisy or outlier samples in small datasets. In contrast, SVM with an RBF kernel underfit the data (R^2^=0.846), suggesting limited capacity to capture the intrinsic non-linear relationships within the 21D feature space. KNN (R^2^=0.717) and a fully connected Neural Networks (NN, R^2^=0.618) suffered from severe overfitting: as a lazy learner, KNN lacks inductive bias for generalization, while the over-parameterized NN memorized dataset-specific noise rather than extracting transferable structure-property rules—a well-documented challenge in small-sample materials informatics.^[4]^

Residual analysis of the RF model (Figure S4f) reveals that >95% of prediction lie within a ±1.0% absolute error band relative to experimental PCE values, with residuals exhibiting homoscedasticity (uniform variance) across the full efficiency range. A minor systematic overestimation is observed, which we attribute not to algorithmic bias, but to an inherent characteristic of our descriptor design: Molecular features are derived from idealized structures and thus reflect the theoretical passivation potential, whereas experimental PCEs are inevitably limited by real-world fabrication imperfections (e.g, film morphology, interfacial contamination). Consequently, this consistent offset does not affect the model's reliability in relative ranking or high-throughput screening, where predictive ordering rather than absolute accuracy is paramount.


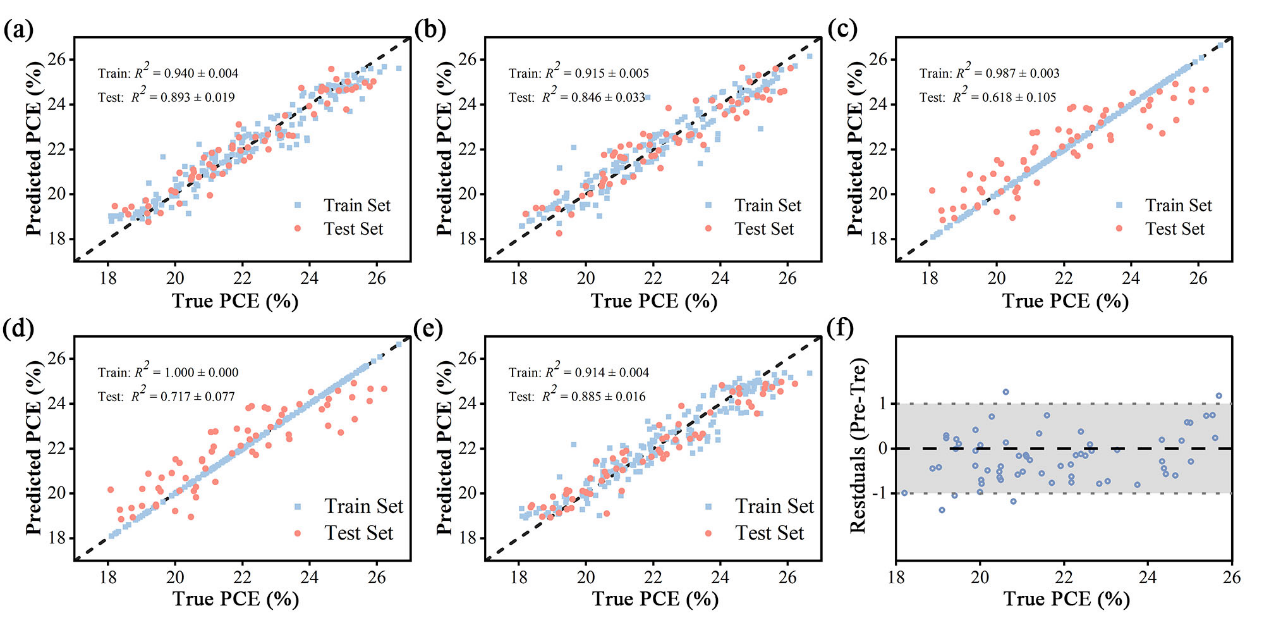


**Figure S4.** Performance evaluation of various ML models. Parity plots comparing the performance of different ML models: (a)XGBoost, (b) SVM, (c) NN, (d) KNN, and (e) LightGBM. Each subplot displays prediction for the test set with dada points representing true vs. predicted PCE values. The insets show the mean test R^2^±standard deviation over 10 runs. f) Residual analysis for the optimal RF model on the test set. Residuals are defined as Predicted−True PCE. The gray band indicates a ±1.0% error margin, highlighting the precision of predictions.

Prior to SHAP analysis, we conducted an initial feature ranking using the built-in Random Forest importance metric (based on MSE impurity reduction). This step was intended not to infer mechanistic insights relationships, but rather to assess the overall relevance and utilization of our engineered feature set by the model. .

As shown in **Figure S5**, the ranking aligns well with physical intuition. I_PCE ranks highest (importance score≈0.72), underscoring its dominant role as a proxy for baseline device quality. Among molecular and system-level descriptors, rA, gmean, LogP, TPSA, and SHBd also exhibit high importance. This confirms that the model effectively leverages our physically grounded features—designed to encode steric, electronic, and intermolecular bonding properties. However, since RF feature importance is agnostic to the direction of influence (i.e., whether a feature enhances or surpasses performance), it cannot resolve nuanced structure-property relationships.


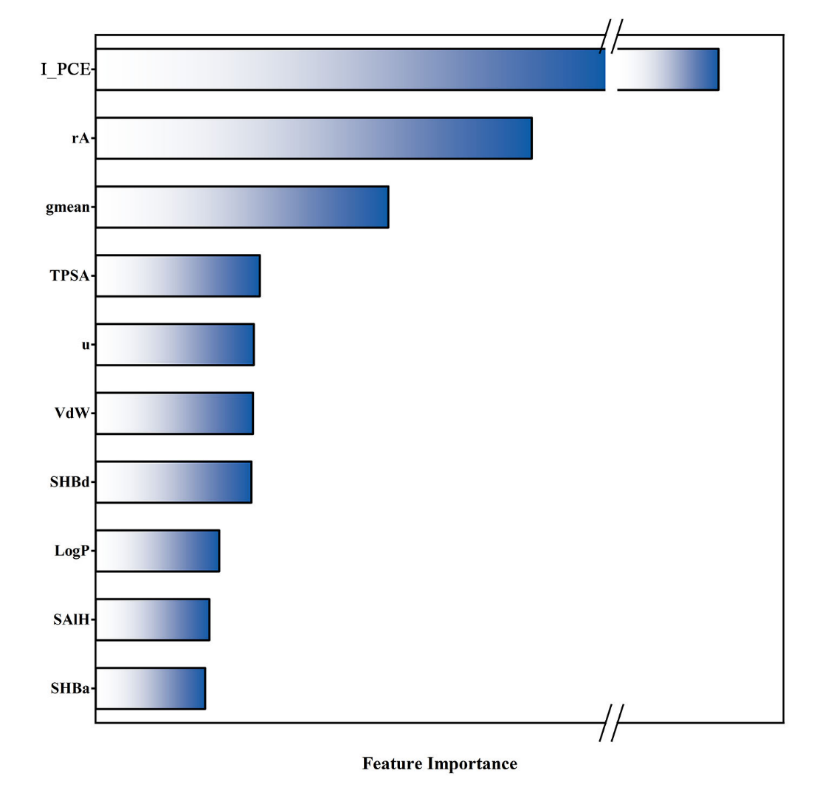


**Figure S5.** Random Forest feature importance ranking based on the MSE impurity criterion. Feature importance ranking for the RF model, calculated using the mean decrease in MSE criterion. The top 15 features are displayed, ranked by their relative importance. Importance scores are normalized to sum to 1 for ease of comparison. Note the break in the *x*-axis to effectively visualize features with lower importance compared to I_PCE, which is the most significant feature.

**Table S4.** Comparative performance of six regression algorithms trained on the full 21-feature set with hard sample mining. Reported values represent mean±standard deviation across 10 runs.

| Model | Train-R^2^ | Test-R^2^ | Train-MAE | Test-MAE | Train-RMSE | Test-RMSE |
| --- | --- | --- | --- | --- | --- | --- |
| RF | 0.940 ± 0.002 | 0.914 ± 0.007 | 0.381 ± 0.005 | 0.520 ± 0.035 | 0.520 ± 0.001 | 0.626 ± 0.033 |
| XGBOOST | 0.940 ± 0.004 | 0.893 ± 0.019 | 0.402 ± 0.013 | 0.570 ± 0.048 | 0.522 ± 0.013 | 0.690 ± 0.057 |
| SVM | 0.915 ± 0.005 | 0.846 ± 0.033 | 0.496 ± 0.011 | 0.672 ± 0.050 | 0.624 ± 0.011 | 0.816 ± 0.066 |
| NN | 0.987 ± 0.003 | 0.618 ± 0.105 | 0.127 ± 0.014 | 1.005 ± 0.104 | 0.237 ± 0.027 | 1.318 ± 0.168 |
| KNN | 1.000 ± 0.000 | 0.717 ± 0.077 | 0.000 ± 0.000 | 0.895 ± 0.095 | 0.000 ± 0.000 | 1.091 ± 0.119 |
| LightGBM | 0.914 ± 0.004 | 0.885 ± 0.016 | 0.472 ± 0.013 | 0.592 ± 0.044 | 0.623 ± 0.011 | 0.719 ± 0.046 |

**Note S5. Supplementary Discussion on SHAP Interpretability Analysis**

In the main text, we employed the SHAP (SHapley Additive exPlanations) method to perform a rigorous, interpretable dissection of the model’s predictions. This approach was chosen to address key limitations of conventional impurity-based feature importance (Figure S5), which—while useful for assessing overall relevance—fails to capture the direction (positive or negative) of feature effects and cannot resolve nonlinear interactions among variables. SHAP analysis overcomes these shortcomings by providing quantitative, sampling-wise attribution of each feature’s contribution to the predicted outcome. The global SHAP summary plot (**Figure S6**) provides a holistic view of feature impacts across the entire dataset, confirming that experimental baseline parameters—most notably I_PCE—exert the strongest influence on model output. This dominance underscores the necessity of our decoupling strategy, which isolates intrinsic molecular contribution from platform-dependent effects, as detailed in the main text.

To uncover non-linear independencies among molecular attributes, we examined SHAP dependence plots for key feature pairs (**Figure S7**). The interplay between electronic structure and steric bulk—quantified by gmean and vdW—reveals a nuanced design principle (Figure S7a): While pronounced charge inhomogeneity (high gmean) is essential for high PCE, peak performance is achieved only when steric bulk remains moderate to low. Highly compact molecules (low vdW) underperform, indicating that an optimal passivator must strike a balance between strong electron-donating character and sufficient conformation flexibility to minimize steric hindrance at the perovskite interface. A striking "switch-like" behavior emerges in the relationship between hydrogen bonding and hydrophobicity (SHBa vs. LogP, Figure S7b). Positive contributions from hydrogen bond acceptors (high SHBa) occur almost exclusively in hydrophobic environments (higher LogP), whereas the same functional groups in hydrophilic contexts tend to degrade performance. This implies that the efficacy of defect anchoring sites is environment-dependent: Within a hydrophobic scaffold, acceptors effectively coordinated undercoordinated Pb^2+^ ions, but in hydrophilic settings, they may inadvertently attract moisture, accelerating device degradation. The coupling between molecular rigidity and polarity (RBR vs. u, Figure S7c) further reveals that a large dipole is detrimental in rigid molecules, likely due to fixed unfavorable electrostatic orientations. However, this penalty is mitigated with increasing flexibility (higher RBR), as conformational freedom enables adaptive reorientation of the dipole to avoid repulsive interactions with the perovskite surface. Finally, the synergy between electrostatic asymmetry and field strength (uD vs. EPD, Figure S7d) identifies an optimal configuration: Maximizing the electrostatic potential difference (EPD) on a scaffold exhibiting significant charge asymmetry (high uD). This combination fosters a robust interfacial electric field that enhances carrier separation and suppresses recombination.

We further leveraged SHAP dependence plots to dissect the intricate interplay between passivation efficacy and perovskite experimental system parameters (**Figure S8**). The analysis reveals that the influence of key compositional variables—rA, B:X, and A:X—on device performance is highly non-monotonic and strongly modulated by the initial film quality I_PCE. For the A-site cation radius (rA, Figure S8a), positive contributions to PCE are confined to a narrow window of 2.4 Å to 2.6 Å, aligning with the theoretical optimal range of the Goldschmidt tolerance factor in stable perovskite.^[5]^ Crucially, this benefit is conditional on high initial film quality: Only devices with high I_PCE (red dots) exhibit performance gains from precise rA tuning, whereas in lower-quality films (blue dots), even minor deviations from this range lead to sharp performance deterioration. Regarding the B:X (Pb:X) molar ratio (Figure S8b), our model provides quantitative support for the widely adopted "Pb-rich" strategy. A moderate Pb excess (B:X≈0.32-0.34) consistently enhances performance across the dataset. However, the consequence of deviation from this optimum are quality-dependent: In high-quality films, excessively Pb becomes the dominant limiting factor, likely due to metallic Pb^0^ formation or trap clustering; in contrast, low-quality films suffer more severely insufficient Pb, which exacerbates halide vacancy concentrations and interfacial recombination. For the A:X molar ratio (Figure S8c), the direction of its effect is almost entirely governed by I_PCE. In high-quality films, increasing A:X yields clear benefits—persumably by passivating surface undercoordinated Pb^2+^ sites. Collectively, in low-quality films (blue dots), any adjustment to A:X is generally detrimental. We attribute this to accumulation of excess organic cations at disordered grain boundaries, which impedes charge transport and amplifies non-radiative losses. ^[6,7]^ Collectively, these findings demonstrate that the optimal compositional tuning is not universal but context-dependent: The same parameter can be beneficial, neutral, or harmful depending on the underlying crystallinity and defect landscape of the perovskite film. This underscores the necessity of adaptive process optimization aligned with intrinsic film quality—a principle critical for reproducible high-performance device fabrication.

Furthermore, to quantitatively validate the predictive boundaries suggested by the global SHAP analysis, we conducted a multi-stage ablation study (**Table S5**). Integrating all features (Model A) achieves maximal accuracy. While the four platform descriptors alone (Model C) establish a strong baseline envelope (R^2^=0.807), removing the initial efficiency (Model B) still maintains robust performance (R^2^=0.775). Crucially, strictly isolating the 17 molecular descriptors (Model D) yields an R^2^ of 0.602. This definitively confirms that our engineered molecular features possess strong independent predictive validity and capture intrinsic structure-property relationships, rather than merely fitting macroscopic baseline noise.


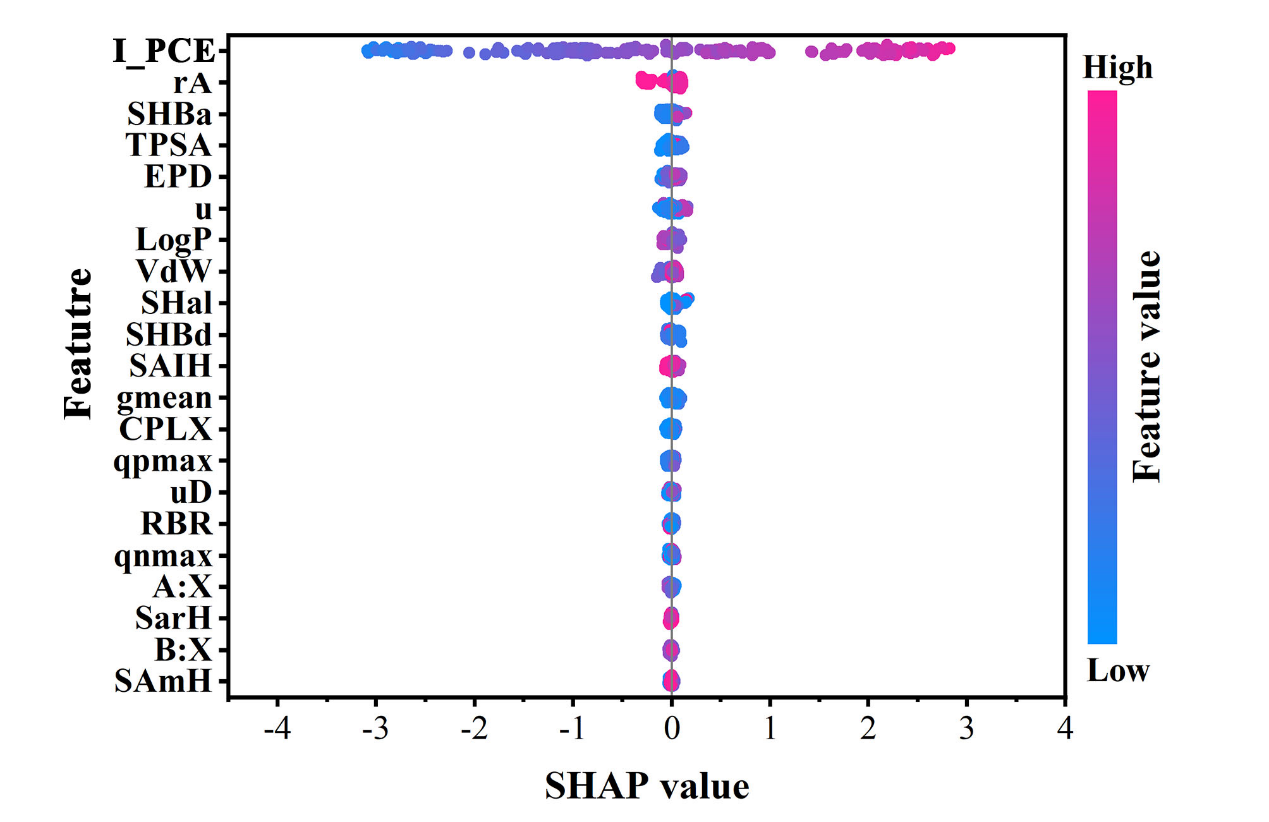


**Figure S6.** Global SHAP analysis including experimental system parameters. Summary plot for all 21 input features, extending the analysis from Figure 3a to include key experimental system parameters: I_PCE, rA, A:X, and B:X. Features are ranked by their global importance (mean |SHAP value|), with I_PCE dominating the model output.

| Model | Feature Set | Feature Count | Test-R^2^ | Test-MAE |
| --- | --- | --- | --- | --- |
| Model A | Comprehensive Model (Platform + Molecular) | 21 | 0.914 ± 0.007 | 0.520 ± 0.035 |
| Model B | Ablated Model  (without I_PCE) | 20 | 0.775 ± 0.002 | 0.740 ± 0.010 |
| Model C | Platform Descriptors Only  (I_PCE, rA, A:X, B:X) | 4 | 0.807 ± 0.025 | 0.727 ± 0.044 |
| Model D | Purely Molecular Descriptors | 17 | 0.602 ± 0.003 | 0.991 ± 0.015 |

Table S5. Quantitative predictive performance of RF models trained on distinct feature subsets.


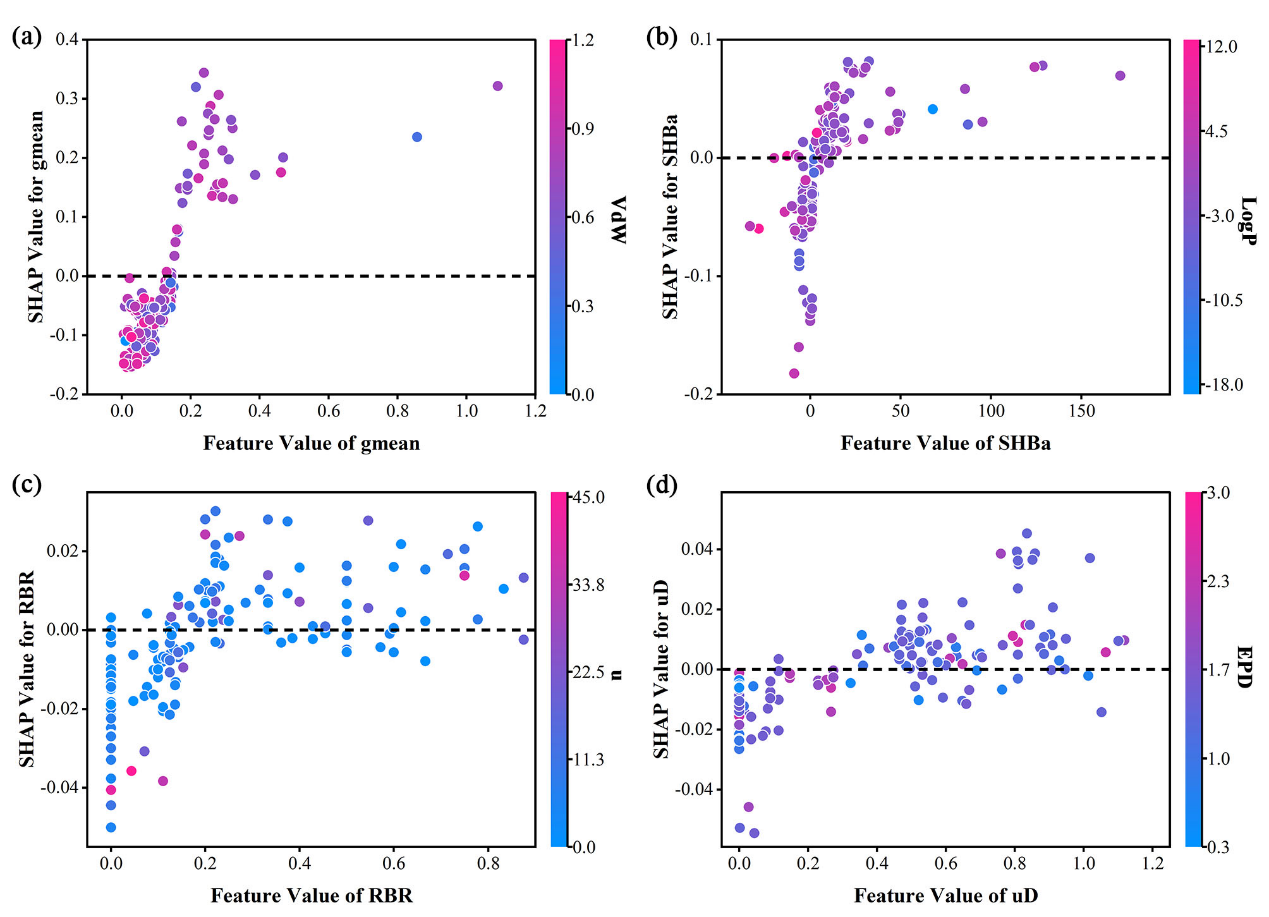


**Figure S7.** SHAP dependence plots illustrating non-linear feature interactions. The plots displaying the interaction effects between key features and their corresponding SHAP values: a) gmean vs vdW, b) SHBa vs LogP, c) RBR vs u, and d) uD vs EPD. For each plot, the *x*-axis represents the primary feature value, the *y*-axis shows its corresponding SHAP value, and the color scale indicates the value of the interacting feature, highlighting how changes in one feature affect the SHAP value of another.


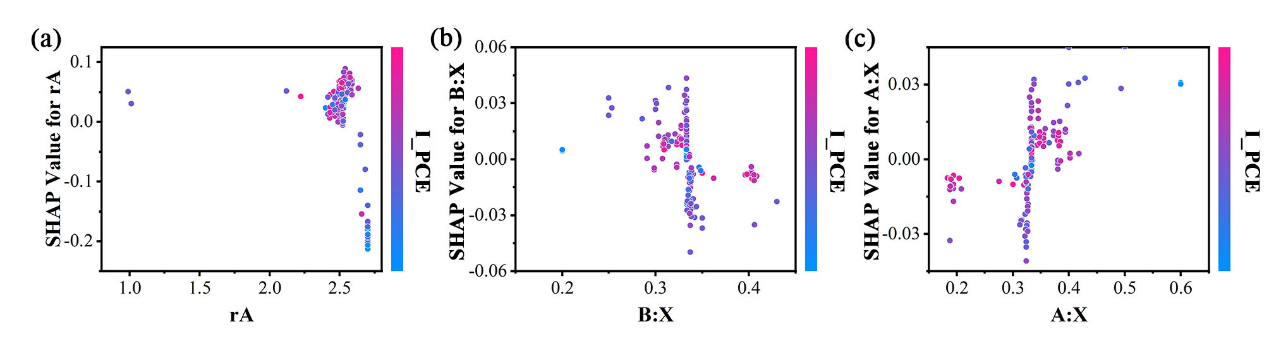


**Figure S8.** SHAP dependence analysis for experimental system parameters. The plots illustrating the impact of key experimental parameters on PCE prediction: a) rA (A-site cation radius), b) B:X (Pb:X molar ratio), and c) A:X (A:X molar ratio). For each plot, the *x*-axis represents the value of the primary feature (e.g., r_A_, B:X, A:X); The *y*-axis shows the corresponding SHAP value, indicating the impact of the feature on PCE predictions.

**Note S6. Detailed Workflow and Criteria for High-Throughput Virtual Screening**

We implemented a hierarchical virtual screening workflow (Figure 4a) to systematically identify promising perovskite passivators from the PubChem database (>121 million compounds). The three-stage pipeline progressively refines the chemical space, ensuring that final candidates exhibit high intrinsic passivation potential, favorable physicochemical properties, and practical synthetic feasibility.

The first stage—formula-based pre-screening—applied computationally light-weight filters derived solely from molecular formulas. Molecular weight (MW) was constrained to 50-600 Da: The lower bound excludes highly volatile species incompatible with solution-based film processing (notably below the minimum MW of 63.06 Da in our training set), while the upper bound ensures coverage of >75% of known high-performance passivators while excluding sterically hindered macro-molecules that are unlikely to access interfacial defect sites.

The degree of unsaturation (DoU) was limited to 1.0-20.0 to eliminate both fully saturated acyclic aliphatic chains (low DoU)—which lack directly binding motifs—and excessively complex polycyclic architectures (very high DoU) that may compromise solubility or processability. Additionally, elemental composition filters were imposed to enrich for functional rich scaffolds: Heteroatom-to-carbon (X/C)≥0.1, ensuring sufficient density of N, O, S, or halogen atoms for defect coordination; Hydrogen-to-carbon (H/C)≤2.2), favoring π-conjugated or aromatic systems over aliphatic hydrocarbons. This criterion collectively reduced the initial library from 121,444,256 to 102,697,501 molecules (~85% retention), establishing a chemically diverse yet tractable starting pool for subsequent structure-aware filtering.

The second stage employed structure-based motif screening using SMILES representations to reinforce functional and chemical design principles. First, a "structural alert" filter—implemented via SMARTS patterns—to systematically excluded molecules containing unstable or highly reactive groups (e.g., peroxides, acyl halides), ensuring baseline chemical stability under device processing conditions. More critically, we enforced a "dual-functional" constraint grounded in the mechanistic understanding that effectively passivators must simultaneously address Lewis acidic and Lewis basic defects. To this end, we curated two complementary libraries of SMARTS patterns: One encoding electron-donating motifs, and the other capturing electron-acceptor groups. Only molecules matching at least one pattern from each library were retained—a strict logical "AND" condition that prioritizes candidates capable of multivalent, bidirectional defect anchoring. This step reduced the pool to 1,376,927 structurally stable, functionally balanced candidates.

In the final stage—feature-based optimization—we applied quantitative constraints on 2D molecular features calculated for the remaining ~1.38 million compounds. Thresholds were rigorously derived from SHAP-guided insights and statistical distributions in our training set to ensure relevance to high-performance passivation Topological Polar Surface Area (TPSA) restricted to 20.0-140.0 Å^2^ and Partition Coefficient (LogP) to –3.0-3.5. Together, these ensure an optimal balance between polarity (for defect interaction) and hydrophobicity (for moisture resistance). To favor molecules of moderate size and conformational rigidity, Heavy Atom Count (HAC) was limited to 5-50 and Rotatable Bond Fraction (RBF) capped at 0.35. Synthetic accessibility was addressed by limiting Ring Count to 1-5 and Chiral Centers to≤2. Finally, fluorine count was capped at 6 to mitigate solubility challenges and aggregate risks associated with excessive fluorination. This multi-objective filtering yielded a high-quality candidate library of 789,931 molecules, optimized for passivation efficacy, environmental stability, and practical synthesizability—ready for downstream ML prediction and experimental validation.

**Note S7. Prediction Uncertainty Quantification via Quantile Regression**

In our virtual screening workflow, the "PCE Improvement Ratio"—defined as the predicted final PCE divided by the baseline device efficiency (I_PCE)—serves as the primary evaluation metric. Initial modeling efforts using standard mean regression revealed that accurate point prediction of this ratio is inherently challenging, yielding only modest out-of-sample R^2^ scores (~0.59), likely due to the complex interplay between molecular structure, processing conditions, and defect physics. Recognizing the limitations of point estimates in high-uncertainty regimes, we adopted a probabilistic screening paradigm: Rather than relying on a single potentially biased prediction, we prioritize reliable prediction intervals that bound that plausible performance range of each candidate.

To implement this, we implemented quantile-based uncertainty quantification leveraging the ensemble architecture of Random Forests. Specifically, we trained a RandomForestRegressor with 200 decision trees. For any input molecule, the distribution of predictions across individual trees approximates the empirical posterior of the target ratio. From this distribution, we extracted the 5th (q_0.05_) and 95th (q_0.95_) percentiles to construct a data-driven 90% prediction interval, with the ensemble mean serving as the central estimate.

Validation on an independent test set (70:30 train-test split) demonstrated the robustness of this approach (**Figure S9**). While the mean prediction achieved an R^2^ of 0.59—reflecting the intrinsic difficulty of precise ratio forecasting—the 90% prediction intervals captured the experimental values in 94.44% of test cases. This near-nominal coverage rate (slightly conservative, as expected) demonstrates that our model reliably "bracket" the actual performance potential of unseen molecules. Critically, this capability enables risk-aware candidate prioritization: instead of selecting solely based on optimistic point estimates, we can identify molecules whose entire plausible performance envelope exceeds a desired threshold. This strategy underpins the final selection protocol described in the main text, ensuring that top-ranked candidates are not only promising but also robust to predictive uncertainty.


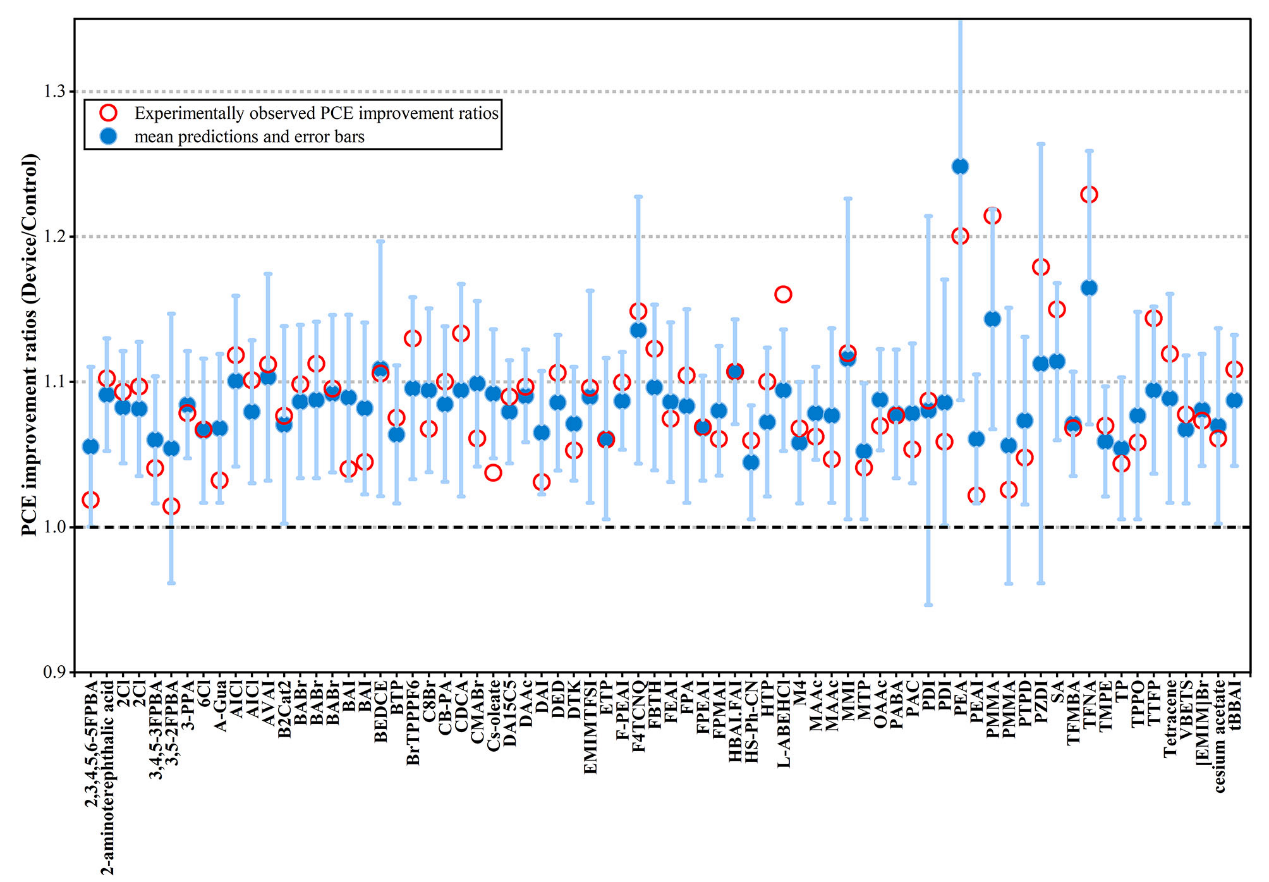


**Figure S9.** Validation of the Random Forest Quantile Regression model on the test set. Comparison of experimentally observed PCE improvement ratios (open red circles) with predictions from RF model: Solid blue dots represent the expected values (mean prediction) from the model, and light blue error bars denote the 90% prediction intervals (5th to 95th percentile). The model achieves a 94.44% coverage rate, confirming its reliability in bounding the performance range of unseen samples.

**Note S8. Prediction, Ranking, and Final Selection of Screened Molecules**

To evaluate the passivation potential of the 789,931 candidate molecules under realistic and representative experimental conditions, we defined three virtual fabrication scenarios. Each scenario fixes key perovskite system parameters—rA, A:X, B:X—at the mean values observed in the top 5% highest-performing samples from the training set, thereby simulating an optimized processing platform. Concurrently, the baseline device efficiency (I_PCE) was set to the 60th (21.09%), 80th (22.60%), and 95th (23.51%) percentiles of the training distribution, representing low-, medium-, and high-performance starting films, respectively. Using the PCE Improvement Ratio (Predicted PCE/I_PCE) as the primary screening metric, we first identified the top 5,000 molecules in each scenario. The intersection of these three high-performing sets—comprising molecules that consistently excel across all quality regimes—was designated the "Core Robust Set" and directly included in the final candidate pool. The remaining scenario-specific top performers formed the "Scenario-Elite Pool", which underwent a diversity-aware selection protocol to avoid over-presentation of dominant scaffolds.

To ensure structural diversity, we applied a clustering-and-sampling strategy to the Scenario-Elite Pool. Molecular similarity was encoded using Extended-Connectivity Fingerprints (ECFP4), generated via the Morgan algorithm (radius=2), to produce 2048-bit binary vectors that capture atom-centered chemical environment up to two bonds away. These fingerprints were then clustered using Butina algorithm, an efficient "leader-follower" method that groups molecules into "chemical coherent families" based on Tanimoto similarity (threshold=0.65). This approach naturally yields clusters of variable sizes, reflecting the intrinsic heterogeneity of chemical space.

To visualize the global distribution of candidates, we projected the 2048-dimensional fingerprint space into two dimensions using t-Distributed Stochastic Neighbor Embedding (t-SNE) (Figure 4c). By minimizing the Kullback-Leibler divergence between high- and low-dimensional probability distributions, t-SNE preserves local neighborhood structures, revealing distinct chemical neighborhoods within the elite pool. During sampling, only clusters containing ≥10 members were considered to ensure statistical relevance. From each qualified cluster, the top 5 molecules were selected based on an "Overall Rank Score", defined as a weighted sum of scenario-specific rankings (Score=1×Rank_low_ + 2×Rank_mid_ + 4×Rank_high_). This weighting scheme explicitly prioritizes performance in high-efficiency contexts, where marginal gains are most valuable. This procedure yielded 1,201 structurally diverse candidates, which were merged with the "Core Robust Set" to form the final evaluation library.

For these high-quality candidates, we quantified prediction uncertainty in the medium- and high-performance scenarios using the Random Forest Quantile Regression model developed in Note S7. Final selection integrated four criteria: High central tendency (median prediction, q_0.5_), strong lower- bound guarantee (q_0.05_), promising attractive upper bound (q_0.95_), and low prediction uncertainty (narrow 90% prediction interval). Balancing these factors, we identified the five most promising candidates, reported in the main text. Their complete chemical structures, properties, and predicted performance envelopes are provided in **Table S6**.

To rigorously assess the experimental feasibility of the five ML-identified passivators (TDZ-S, TZC-F, TZC-P, DZP-A, ODZ-F), we conducted automated retrosynthetic analysis using ChemAIRS, an AI-powered synthesis planning platform. ChemAIRS integrates large-scale reaction data mining with deep learning to rapidly propose viable synthesis routes—typically within minutes—while explicitly evaluating reaction feasibility, functional group compatibility, and stereochemical control. For each candidate, ChemAIRS generated multiple plausible pathways. we then applied a stringent set of practical criteria to select the optimal route: Exclusion of high-energy, toxic, or known allergenic intermediates; Avoidance of hazardous reagents or extreme reaction conditions (e.g., high-pressure, pyrophoric reagents); Minimal synthetic length (≤4 steps); Low estimated material cost, based on precursor availability and scalability. The resulting optimized routes (**Figure S10**) demonstrate that all five molecules can be synthesized in ≤4 steps from commercially available building blocks using well-established organic transformations (e.g., nucleophilic substitution, Suzuki coupling, reductive amination). Critically, no exotic reagents or specialized equipment are required. This confirms not only the synthetic accessibility but also the practical deployability of these candidates for scalable perovskite device fabrication.

**Table S6.** Detailed chemical information of the five final candidate passivators screened in this study.

| Abbrevia-tion | | PubChem CID | Molecular Formula | IUPAC Name |
| --- | --- | --- | --- | --- |
| TDZ-S | 112752105 | | C_9_H_18_F_3_N_3_O_2_S | N-[2-(1,1-dioxo-5-propyl-1,2,5-thiadiazolidin-2-yl)ethyl]-2,2,2-trifluoroethanamine |
| TZC-F | 114383479 | | C_7_H_11_F_2_N_3_O_2_S | N-(3-amino-2,2-difluoropropyl)-2-oxo-1,3-thiazolidine-4-carboxamide |
| TZC-P | 119507059 | | C_13_H_26_C_l_N_3_O_2_S | 3-(2,2-dimethylpropanoyl)-N-[2-(ethylamino)ethyl]-1,3-thiazolidine-4-carboxamide hydrochloride |
| DZP-A | 119844036 | | C_15_H_32_C_l2_N_4_O_2_ | 2-[4-(2-amino-2-methylpropanoyl)-1,4-diazepan-1-yl]-N,N-diethylacetamide dihydrochloride |
| ODZ-F | 68757163 | | C_7_H_13_C_l_FN_3_O | 2-[5-(2-Fluoropropan-2-yl)-1,2,4-oxadiazol-3-yl]ethanamine hydrochloride |

**
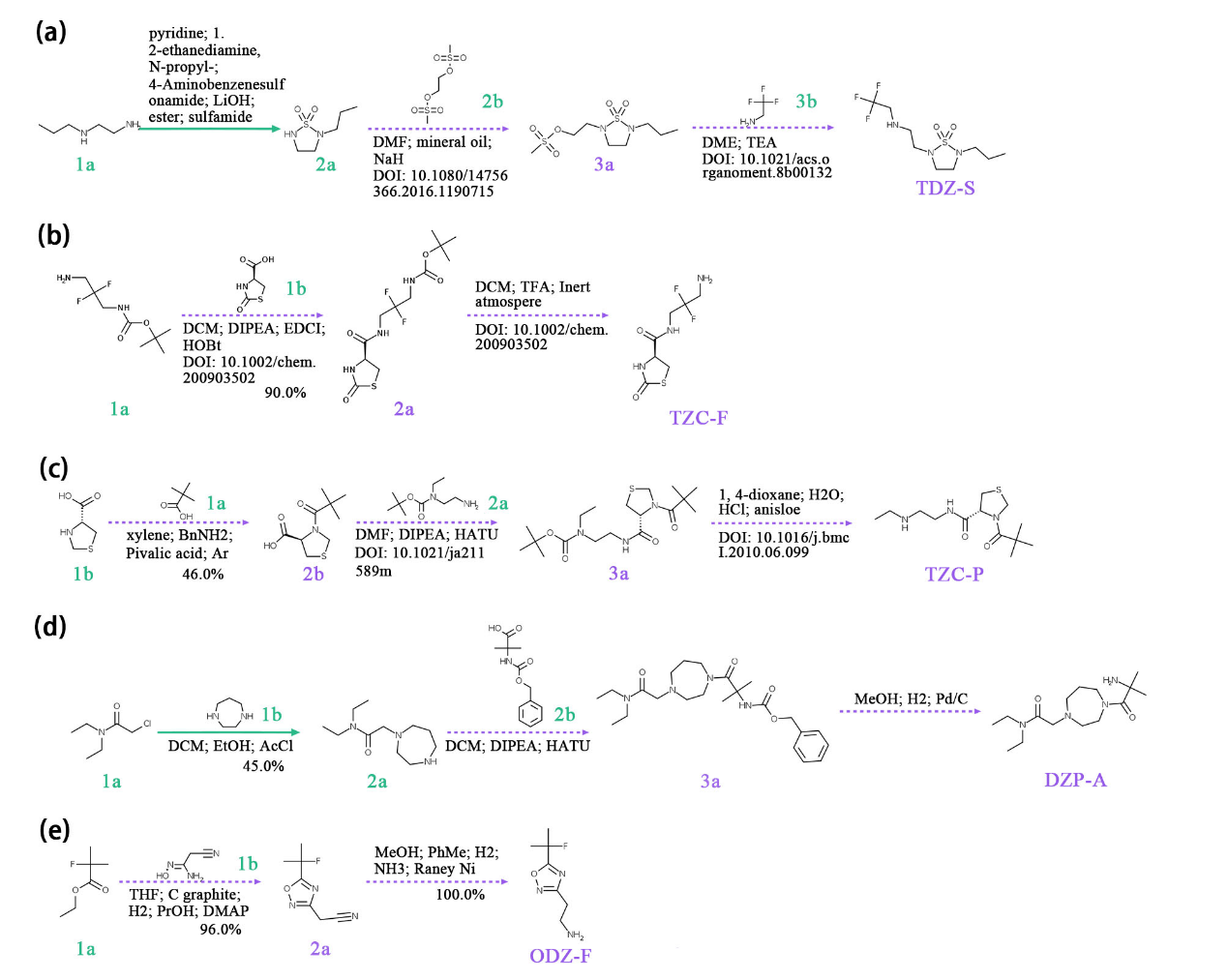
**

**Figure S10.** Retrosynthetic routes for the five finalist candidates predicted by ChemAIRS. a) TDZ-S, b) TZC-F, c) TZC-P, d) DZP-A, and e) ODZ-F. Green labels/arrows indicate commercially available starting materials or established reaction pathways, while purple labels/arrows represent predicted intermediates and key synthetic steps. All targets can be synthesized within 2–4 steps from readily available precursors, utilizing well-established organic transformation such as nucleophilic substitution, Suzuki coupling, and reductive amination.

**Note S9. First-Principles Computational Methods**

To elucidate the atomic- and electronic-level origins of the passivation performance of the five ML-identified candidates—and to provide a theoretical foundation for their interactions with the perovskite surface—we employed a multi-step first-principles computational protocol based on DFT. All molecular-level calculations were performed using Gaussian 16, following two-stage workflow: 1. Unconstrained geometry optimization to locate stable conformers; 2. Electronic structure analysis, including electrostatic potential (ESP) mapping, based on the optimized geometries. For geometry optimization, we utilized the B3LYP (Becke, 3-parameter, Lee-Yang-Parr) hybrid functional with the 3-21G basis set—a well established level of theory that offers an optimal trade-off between computational efficiency and accuracy for medium-sized organic molecules. Calculations were performed in the gas phase, without symmetry constraints, to allow full conformational flexibility. The opt keyword was used for energy minimization, while freq enabled vibrational frequency analysis to confirm that all final structures correspond to true minima on the potential energy surface (i.e., exhibiting no imaginary frequencies). To preserve the correct bonding topology throughout optimization, the geom=connectivity keyword was applied, ensuring consistency with initial molecular connectivity. Subsequently, ESP was computed at the same B3LYP/3-21G level and visualized it using GaussView 6 by projecting it onto an isosurface of the total electron density (isovalue=0.002 a.u.), as shown in Figure 5a of the main text. This ESP mapping highlights region of negative electrostatic potential (electron-rich sites), which serve as Lewis basic centers or hydrogen-bond acceptors, and positive potential region (electron-deficient sites), corresponding to Lewis acidic or hydrogen-bond donors. These spatially resolved electrostatic features provide critical guidance for predicting favorable adsorption configurations on perovskite surfaces—particularly at undercoordinated Pb^2+^ (Lewis acid) and halide vacancy (Lewis base) defect sites.

To model the molecular-level interaction between top-performing passivators and the perovskite surface, we performed periodic DFT calculations using the Vienna Ab-initio Simulation Package (VASP) 5.4.4. Electrons-ion interactions were treated with the Projector Augmented Wave (PAW) method, and the exchange-correlation effects were described by the Perdew-Burke-Ernzerhof (PBE) functional within the Generalized Gradient Approximation (GGA). The computational model was built upon the cubic β-phase CsPbI_3_. We first fully relaxed the bulk structure and then cleaved it along the [001] direction to construct a four-layer PbI_2_ terminated slab. To mimic an isolated surface, a 20 Å vacuum layer was inserted perpendicular to the slab (along the *z*-axis), effectively suppressing spurious inter-slab interactions under periodic boundary conditions. During structural optimization, the bottom two atomic layers were fixed at their bulk-equilibrium positions to emulate the influence of the underlying crystal, while the top two atomic layers and any adsorbed molecule were fully relaxed. Given the intrinsic polarity of PbI_2_-terminated surface, dipole correction was applied along the *z*-direction to eliminate artificial electrostatic artifacts.

Prior to interface modeling, each passivator molecule was pre-optimized in isolation within a 30 Å×30 Å×30 Å cubic supercell, ensuring negligible interaction between periodic images. These gas-phase optimizations employed: A plane-wave energy cutoff (ENCUT) of 250 eV; Gamma point (1×1×1 *k*-mesh) for Brillouin zone integration; An electronic convergence threshold of 1×10^−5^ eV; The Conjugate Gradient (CG) algorithm for ionic relaxation. Structures were considered converged when forces on all atoms were less than 0.02 eV/Å, with dipole corrections enabled in all directions.

For the adsorption simulations, the pre-optimized molecules were placed on the relaxed β-CsPbI_3_(001) surface. Computational setting were kept consistent with those of the clean slab. Guided by ESP maps from Gaussian-based molecular analysis (Figure 5a), we designed multiple initial adsorption configurations for each candidate—systematically exploring "parallel" vs "vertical" orientations and evaluating both single-point and multi-point binding motifs involving Lewis basic sites (e.g., N, O, S) interacting with undercoordinated Pb^2+^ or Lewis acidic sites engaging with I^-^ vacancies. After full relaxation of each adsorption complex, the binding energy (E_bind_) was computed as E_bind_=E_total_ − (E_slab_ + E_mol_), where E_total_, E_slab_, and E_mol_ represent the total energies of the adsorption system, the pristine slab, and the isolated molecule, respectively; Negative E_bind_ values indicate thermodynamically favorable adsorption.

To assess the impact of molecular adsorption on the surface electronic structure, we computed the electrostatic potential profile across the vacuum region for both pristine and the passivated surfaces. Combined with the Fermi level output by VASP, this allowed us to determine the Work Function. The Valence Band Maximum (VBM) and Conduction Band Minimum (CBM) were then aligned relative to the vacuum level, enabling direct comparison of band-edge shifts induced by passivation.

Finally, to quantify interfacial charge redistribution, we calculated the differential charge density (Δρ) using the formula Δρ=ρ_total_ − (ρ_slab_ + ρ_mol_), where, ρ_total_, ρ_slab_, and ρ_mol_ are the charge densities of the adsorption system, isolated slab, and isolated molecule, respectively—all evaluated with all atomic positions fixed at their final relaxed coordinates. The resulting Δρ was further analyzed via planar averaging and charge displacement curves to visualize the direction and magnitude of the electron transfer at the interface.

**References**

1. A. Shrivastava, A. Gupta, and R. Girshick, “Training Region-Based Object Detectors with Online Hard Example Mining,” *Proceedings of the IEEE Conference on Computer Vision and Pattern Recognition (CVPR)* (2016): 761–769.
   https://doi.org/10.1109/CVPR.2016.89
2. W. Liu, Y. Lu, D. Wei, X. Huo, X. Huang, Y. Li, J. Meng, S. Zhao, B. Qiao, Z. Liang, Z. Xu, and D. Song, “Screening Interface Passivation Materials Intelligently through Machine Learning for Highly Efficient Perovskite Solar Cells,” *Journal of Materials Chemistry A* 10 (2022): 17782-17789. https://doi.org/10.1039/D2TA04788H
3. C. Zhi, S. Wang, S. Sun, C. Li, Z. Li, Z. Wan, H. Wang, Z. Li, and Z. Liu, “Machine-Learning-Assisted Screening of Interface Passivation Materials for Perovskite Solar Cells,” *ACS Energy Letters* 8 (2023): 1424–1433.
   https://doi.org/10.1021/acsenergylett.2c02818
4. L. Grinsztajn, E. Oyallon, and G. Varoquaux, “Why Do Tree-Based Models Still Outperform Deep Learning on Typical Tabular Data?,” *Advances in Neural Information Processing Systems* 35 (2022): 507–520.
   https://doi.org/10.5555/3600270.3600307
5. Z. Li, M. Yang, J.-S. Park, S.-H. Wei, J. J. Berry, and K. Zhu, “Stabilizing Perovskite Structures by Tuning Tolerance Factor: Formation of Formamidinium and Cesium Lead Iodide Solid-State Alloys,” *Chemistry of Materials* 28 (2016): 284–292.
   https://doi.org/10.1021/acs.chemmater.5b04107
6. D.-H. Kang and N.-G. Park, “On the Current–Voltage Hysteresis in Perovskite Solar Cells: Dependence on Perovskite Composition and Methods to Remove Hysteresis,” *Advanced Materials* 31 (2019): e1805214.
   https://doi.org/10.1002/adma.201805214
7. Q. Chen, H. Zhou, T.-B. Song, S. Luo, Z. Hong, H.-S. Duan, L. Dou, Y. Liu, and Y. Yang, “Controllable Self-Induced Passivation of Hybrid Lead Iodide Perovskites toward High Performance Solar Cells,” *Nano Letters* 14 (2014): 4158–4163.
   https://doi.org/10.1021/nl501838y
